# Rational Materials and Structure Design for Improving the Performance and Durability of High Temperature Proton Exchange Membranes (HT‐PEMs)

**DOI:** 10.1002/advs.202303969

**Published:** 2023-08-31

**Authors:** Jingnan Song, Wutong Zhao, Libo Zhou, Hongjie Meng, Haibo Wang, Panpan Guan, Min Li, Yecheng Zou, Wei Feng, Ming Zhang, Lei Zhu, Ping He, Feng Liu, Yongming Zhang

**Affiliations:** ^1^ School of Chemistry and Chemical Engineering Frontiers Science Center for Transformative Molecules Center of Hydrogen Science Shanghai Key Lab of Electrical Insulation & Thermal Aging Shanghai Jiao Tong University Shanghai 200240 P. R. China; ^2^ Shanghai Maxim Fuel Cell Technology Company Shanghai 201401 P. R. China; ^3^ State Key Laboratory of Fluorinated Functional Membrane Materials and Dongyue Future Hydrogen Energy Materials Company Zibo Shandong 256401 P. R. China

**Keywords:** high‐temperature proton exchange membranes, degradation mechanisms, regulation strategies for performance and durability, morphology characterization techniques

## Abstract

Hydrogen energy as the next‐generation clean energy carrier has attracted the attention of both academic and industrial fields. A key limit in the current stage is the operation temperature of hydrogen fuel cells, which lies in the slow development of high‐temperature and high‐efficiency proton exchange membranes. Currently, much research effort has been devoted to this field, and very innovative material systems have been developed. The authors think it is the right time to make a short summary of the high‐temperature proton exchange membranes (HT‐PEMs), the fundamentals, and developments, which can help the researchers to clearly and efficiently gain the key information. In this paper, the development of key materials and optimization strategies, the degradation mechanism and possible solutions, and the most common morphology characterization techniques as well as correlations between morphology and overall properties have been systematically summarized.

## Graphical abstract

High‐temperature proton exchange membrane fuel cells (HT‐PEMFCs) have become the new trend for future development due to the unique advantages. The major obstacle for HT‐PEMFCs is the sluggish development of high‐performance HT‐PEMs. This work systematically summarizes the development of key materials, the degradation mechanisms, the optimization strategies, and the most common morphology characterization techniques.

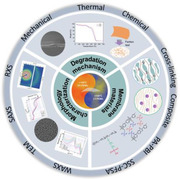

## 1 Introduction

Nowadays, energy and environment are of global concern, and thus researchers have been driven to develop clean, efficient, and sustainable energy technologies. Among them, proton exchange membrane fuel cells (PEMFCs) using hydrogen as fuel have attracted great attention due to their high power density, zero‐emission, and broad range of applications. At present, the PEMFCs on the market are mostly based on perfluorosulfonic acid (PFSA)‐based membranes,^[^ ^1^, ^2^ ^]^ and the optimal operation conditions are at low temperature (<100 °C) and high humidity (>40% RH).^[^ ^3^, ^4^ ^]^ For the commercial PEMFCs, there are some serious challenges:^[^ ^5^ ^]^ 1) Water is the necessary media for the proton transfer, but excessive amount of water could lead to flooding and hinder oxygen transport. Therefore, serious water management is of great significance, and such demands complicate the flow plate structure and the humidification system. 2) The sluggish oxygen reduction reaction process (ORR) at the low temperature needs to be compensated by the abundant Pt catalysts, thus increasing the costs. 3) The poor tolerance of Pt catalysts to CO requires pure H_2_ with CO content below 10 ppm, thus increasing the complexity of fuel processing systems. Such challenges motivated the emergence and development of high‐temperature PEMFCs (HT‐PEMFCs). It is generally accepted that the ORR rate can be significantly improved by elevating the operation temperature above 100 °C, making it possible to develop the high‐performance PEMFC based on low Pt loadings or non‐Pt catalysts.^[^ ^6^ ^]^ As for the problem surrounding CO tolerance, a concentration of 10 ppm significantly poisons the Pt catalysts under 80 °C operating conditions, thus causing a large decrease in performance.^[^ ^7^ ^]^ When the operating temperature is increased to above 180 °C, the CO tolerance is increased to 2%‐5%,^[^ ^8^ ^]^ offering an advantage for researchers to develop a simple hydrogen reformer. Besides, the HT‐PEMFCs provide an anhydrous or low humidity environment during the operation, which can avoid water flooding and thus simplify the fuel cell testing systems. These advantages point out the importance of HT‐PEMFCs, becoming the new trend in both academia and industry.

One of the major obstacles to the development of HT‐PEMFCs is the production of high‐efficient high‐temperature proton exchange membranes (HT‐PEMs). For the HT‐PEMs, they must satisfy the following requirements:^[^ ^9^ ^]^ 1) high proton conductivity under low humidity or anhydrous environment, 2) high glass transition temperature (*T*_g_) to prevent the polymer from becoming viscoelastic at high temperature and losing the necessary mechanical strength, 3) high chemical stability to prevent the chemical degradation under the attack of radicals. However, there are still great challenges for the state‐of‐the‐art PEMs to meet all the requirements simultaneously. For the commercial PFSA membranes, the proton conductivity is highly dependent on the humidity and thus determined that the commercialized PEMFCs are generally operated at relatively low temperatures (70–90 °C) and high humidity conditions (>40% RH).^[^ ^10^, ^11^ ^]^ By regulating the side chain structure^[^ ^12^, ^13^ ^]^ or introducing the hydrophilic proton carries,^[^ ^14^, ^15^, ^16^, ^17^, ^18^ ^]^ the operating temperatures have been expanded to 100–120 °C, but most work is limited at the laboratory level. In 2023, Toyota developed the third generation of PEMFC stacks using the modified PFSA membranes, which can work efficiently and stably at 105 °C. The emergence of Mirai 3 marks a significant breakthrough of HT‐PEMFCs in commercialization. However, the operating temperature is still low, which is not sufficient to satisfy the demands for HT‐PEMFCs. The phosphoric acid‐doped PBI (PA‐PBI) is another type of membrane material, which typically operates at 140–200 °C and anhydrous conditions. The modification of PBI structures,^[^ ^19^, ^20^, ^21^, ^22^, ^23^, ^24^, ^25^ ^]^ cross‐linking treatment,^[^ ^26^, ^27^, ^28^, ^29^, ^30^, ^31^ ^]^ or incorporating protic additives^[^ ^32^, ^33^, ^34^, ^35^, ^36^ ^]^ could improve the proton conductivity and enhance the mechanical/thermal stability, and then improve the performance and durability of HT‐PEMFCs. Nevertheless, the current PA‐PBI membranes are still at the stage of laboratory research, and the corresponding performance and durability of HT‐PEMFCs are still far from the commercial PEMFCs. Besides, the narrow temperature and humidity operating windows further limit the practical application and commercialization of PA‐PBI membranes. Recently, the development of new polymers with strong basic sites located in the backbone or side chains,^[^ ^37^, ^38^, ^39^ ^]^ such as quaternary ammonium (QA)‐biphosphate ion‐pair‐coordinated polyphenylene (QAPOH), pendent imidazole‐functionalized polyphenylene oxide (PPO), Tröger's base (TB)‐based polymers, have successfully broadened the flexibility of working conditions and achieved high stability even under high humidity. These achievements make it possible for the practical application of the PA‐based HT‐PEMs.

This review gives a comprehensive summary of the state‐of‐the‐art HT‐PEMs, the degradation mechanism and possible solutions, and the most common morphology characterization techniques as well as the correlations between morphology and overall properties of PEMs, which are expected to guide future design and development of new material systems and the preparation of high‐quality HT‐PEMs.

## 2 Development of HT‐PEMs

The development of high‐temperature proton exchange membranes (HT‐PEMs) has undergone significant advances in recent years. The key materials for HT‐PEMs are sulfonic acid (SA)‐containing membranes and phosphoric acid (PA)‐containing membranes, which will be discussed in detail in the following section.

### 2.1 Sulfonic Acid (SA)‐Containing Membranes

#### 2.1.1 Modified PFSA membranes

The state‐of‐the‐art Nafion membranes have demonstrated good performance and long durability in the LT‐PEMFCs.^[^ ^40^ ^]^ Despite the success, they are unsuitable for HT‐PEMFCs (>100 °C) due to the dehydration‐induced low proton conductivity and the relatively low glass transition temperature (*T*_g_≈106 °C).^[^ ^10^, ^11^, ^12^ ^]^ To address these issues, various strategies, including chemical structure manipulation,^[^ ^12^, ^13^ ^]^ and additive engineering by incorporating hygroscopic oxides or hydrophilic proton carries,^[^ ^14^, ^17^, ^41^, ^42^ ^]^ have been conducted, which exhibited successful applications in the HT‐PEMFCs.

##### Side‐Chain Manipulation

**Figure 1:**
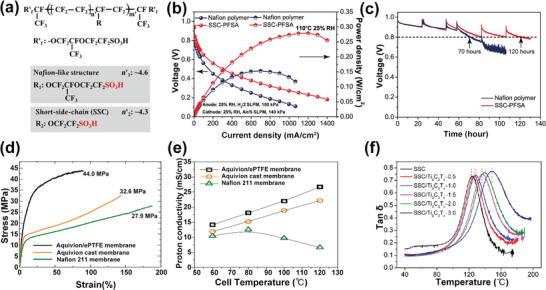
a) The chemical structures of long‐side chain (LSC) PFSA and short‐side chain (SSC) PFSA. b) The polarization performance of PEMs at 110 °C and 25% RH (H_2_/Air). c) The OCV durability test at 90 °C and 30% RH. a‐c) Reproduced with permission.^[^ ^12^ ^]^ Copyright 2023, American Association for the Advancement of Science. d) The strain‐stress curves and e) the proton conductivity of Aquivion/ePTFE composite membrane, Aquivion cast membrane, and Nafion 211 membrane. d,e) Reproduced with permission.^[^ ^53^ ^]^ Copyright 2013, Elsevier. f) The tanδ curves of SSC‐PFSA membranes doped with different amounts of Ti_3_C_2_T_x_. Reproduced with permission.^[^ ^54^ ^]^ Copyright 2021, MDPI.

The first and most obvious approach to improve the performance of HT‐PEMs is to modify the side chain structure. In the 1990s, the Dow Chemical Company first modified the length of side chains and developed the short side chain (SSC) PFSA.^[^ ^43^, ^44^ ^]^ Subsequently, a series of SSC PFSAs with different ionic exchange capacities (IEC) and side chain lengths have been developed, and systematic studies on their physical properties and performances have been conducted.^[^ ^13^, ^45^, ^46^, ^47^ ^]^ Compared with the traditional Nafion membranes, the SSC PFSAs showed an improved *T*_g_, good hydration capacity, and high proton conductivity, which enabled potential applications in moderate temperature (100–120 °C) and low‐relative humidity fuel cells.^[^ ^12^ ^]^ Recently, our research group has prepared homogeneous SSC PFSA membranes and achieved excellent single‐cell performance and durability at high temperatures.^[^ ^12^ ^]^ As shown in **Figure** **1**b,c, the SSC PFSA membrane has a peak power density of 0.279 W cm^−2^ under 110 °C and 25% RH, which is 1.8 times that of the Nafion membrane, and the longer operation lifetime after the open circuit voltage (OCV) durability test indicated the improved chemical stability. Similar good performance was observed in small stack testing with composite SSC PFSA membranes, achieving a maximum power density of 488 mW cm^−2^ at 95 °C and 40% RH.^[^ ^48^ ^]^ Besides experimental research, computational and machine learning research has made significant contributions to the development of PEMFCs.^[^ ^49^, ^50^, ^51^ ^]^ For example, the machine learning results showed that the membrane contributed the most to power density.^[^ ^52^ ^]^ Among various membranes, the Aquivion (E98‐05S) exhibited a relatively larger contribution than that of the Nafion membrane, further confirming that the SSC‐PFSA membrane has a better performance than that of the LSC‐PFSA membrane. Except for the work on improving the performance, there are some works focusing on improving the durability of the SSC‐PFSA membrane. For example, Xiao et al.^[^ ^53^ ^]^ have prepared the Aquivion/ePTFE composite membranes by immersing the ePTFE membranes into the SSC‐PFSA solutions. Compared with the pristine Aquivion membrane, the mechanical strength was significantly improved from 32.6 MPa to 44.0 MPa (Figure 1d) without sacrificing the proton conductivity (Figure 1e). Guan et al.^[^ ^54^ ^]^ have incorporated the 2D MXene, such as Ti_3_C_2_T_x_, into the SSC‐PFSA membranes. With the doping levels from 0 wt% to 3 wt%, the *T*_g_ was elevated from 123 °C to 157 °C (Figure 1f), which is high enough to mitigate the mechanical and chemical degradation during the long‐term operation at 100–120 °C. In the current stage, the research on SSC‐PFSA membranes is still in its infancy, and more work is still needed to promote the SSC‐PFSA membranes to reach the best performance and durability.

##### Additive Engineering

**Figure 2:**
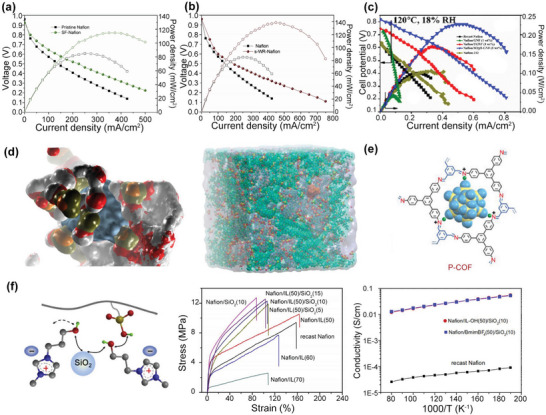
PFSA‐based composite membranes. The polarization performance of a) Nafion/SiO_2_ composite membranes at 110 °C and 20% RH, Reproduced with permission.^[^ ^55^ ^]^ Copyright 2019, Elsevier. b) Nafion/sulfonated SiO_2_ composite membranes at 110 °C and 20% RH, Reproduced with permission.^[^ ^56^ ^]^ Copyright 2019, Elsevier. c) Nafion/SO_3_H‐UGNF at 120 °C and 18% RH. Reproduced with permission.^[^ ^10^ ^]^ Copyright 2021, Elsevier. d) The solvation environment around the HPW molecule and the morphology of Nafion/HPW composite membranes using molecular simulation. Reproduced with permission.^[^ ^70^ ^]^ Copyright 2014, Elsevier. e) The schematic of preparing COF/HPW composite materials to stabilize the HPW molecules. Reproduced with permission.^[^ ^14^ ^]^ Copyright 2023, Elsevier. f) The Nafion/SiO_2_/ILs composite membranes. Left: The Schematic illustration of hydrogen‐bonding interactions in the composite membranes. Middle: The stress‐strain curves of composite membranes. Right: The proton conductivity of composite membranes. Reproduced with permission.^[^ ^67^ ^]^ Copyright 2016, Elsevier.

Although the side chain modification has successfully improved the performance of HT‐PEMs, the relatively lower hydration ability under high‐temperature and low‐humidity conditions still limits the proton conductivity. To further enhance the performance of HT‐PEMs, a series of additives, such as hygroscopic inorganic materials, hydrophilic protic carriers, and their derivatives, have been incorporated into the PFSA‐based membranes to improve the water retention capacity, increase the proton transport channels, and enhance the mechanical strength. SiO_2_, TiO_2_, ZrO_2_, and their derivatives are commonly studied hygroscopic materials.^[^ ^55^, ^56^, ^57^ ^]^ When incorporated into the PFSA‐based membranes, the self‐purification effect during film formation allowed the additives to enter into the PFSA matrix, and the structure was further stabilized by the hydrogen‐bonding interaction between additives and PFSAs. For example, Xu et al. have developed a novel swelling‐filling (SF) strategy and obtained a good SiO_2_/Nafion composite membrane (SF‐Nafion) with SiO_2_ uniformly filling into the Nafion matrix. Compared with the bare Nafion membrane and re‐casted SiO_2_/Nafion (RE‐Nafion) composite membrane, the SF‐Nafion membrane showed enhanced mechanical strength, improved proton conductivity, and good performance of 113 mW cm^−2^ at 110 °C and 20% RH (**Figure** **2**a).^[^ ^55^ ^]^ Further, by the sulfonation of SiO_2_, the synergistic effect of SiO_2_ “water reservoir” and the increased proton conductive channels contributed to higher performance of 140 mW cm^−2^ at 110 °C and 20% RH (Figure 2b).^[^ ^56^ ^]^ Similar sulfonation methods have been applied to functionalize carbon materials, and the most successful examples are Nafion/sulfonated graphene,^[^ ^58^ ^]^ Nafion/sulfonated multiwalled carbon nanotubes,^[^ ^59^ ^]^ Nafion/sulfonated fullerene,^[^ ^60^ ^]^ and Nafion/sulfonated graphitic carbon nitride.^[^ ^61^ ^]^ However, the limited sulfonation sites result in limited performance improvement. To solve this issue, Vinothkannan et al.^[^ ^10^ ^]^ have adopted heteroatom engineering to modulate the graphite nanofibers (GNFs) to layered graphene, which allowed a dense functionalization of ‐SO_3_H groups. The high level of sulfonation significantly improved the proton conductivity and performance and achieved a maximum power density of 230 mW cm^−2^ at 120 °C and 18% RH (Figure 2c).

Hydrophilic proton carrier is another type of additive, which can simultaneously improve water retention and proton conductivity. The representative materials are heteropolyacid (HPA),^[^ ^14^, ^62^, ^63^ ^]^ functionalized polyhedral oligomeric silsesquioxane (POSS) derivatives,^[^ ^15^, ^17^, ^64^ ^]^ and protic ionic liquids (PILs).^[^ ^16^, ^18^, ^41^, ^42^, ^65^, ^66^, ^67^ ^]^ HPAs are MOx polyhedral‐based salts, where the O atoms are easily surrounded by water molecules by forming hydrogen bonds, which are expected to provide more proton transfer sites and increase the proton transfer pathways. At present, the PFSA/HPAs composite membranes have exhibited successful applications in HT‐PEMFCs, and the representative examples are phosphomolybdic acid (HPM), silicotungstic acid (HSW), and phosphotungstic acid (HPW).^[^ ^63^ ^]^ Some theoretical and molecular‐level studies have clarified the mechanism of HTA in improving proton conductivity and cell performance.^[^ ^68^, ^69^, ^70^ ^]^ For example, by combining molecular dynamic (MD) simulations and small‐angle X‐ray scattering (SAXS) experiments, Sambasivarao et al.^[^ ^70^ ^]^ have proposed that the HPW could organize the local water molecules and nearby excess protons to efficiently connect the surrounding proton channels and favor the formation of interconnected proton conducting networks, thus improving the proton conducting characteristics and cell performance (Figure 2d). However, the water‐soluble property of HPAs leads to the composite membrane's instability during fuel cell operation. The ion replacement and chemical bonding have proven to be efficient strategies to stabilize the HPAs.^[^ ^14^, ^62^ ^]^ For example, by ion exchanging protons with larger cations such as Cs^+^, NH_4_^+^, Rb^+,^ and Tl^+^, the solubility of HPW was decreased, hindering the PWA loss from 100% to 5%. The Nafion/modified HPW composite membranes also obtained a good proton conductivity of 16 mS cm^−1^ at 120 °C and 35% RH.^[^ ^62^ ^]^ Zhai et al.^[^ ^14^ ^]^ have used a hydrothermal method to trap the HPW in the cavities of covalent organic framework (COF) through the chemical bonds (Figure 2e), and the single cell based on Nafion/HPW composite membrane exhibited an improved performance and durability at low humidity of 50% RH.

POSS materials have hydrophilic Si─O─Si nano‐cage core structures, and the shell can be functionalized as expected.^[^ ^15^, ^17^, ^64^ ^]^ When incorporated into the PFSA matrix, the POSS fragments can enhance the thermal and mechanical properties through structural reinforcement, and the hydrophilic properties of Si─O─Si structure as well as the proton‐type functional groups can improve the proton conductivity, making it suitable for HT‐PEMFCs. The representative examples are Nafion/ triazole‐POSS,^[^ ^17^ ^]^ Nafion/ImPOSS,^[^ ^15^ ^]^ and Nafion/SO_3_H‐POSS.^[^ ^64^ ^]^ Moreover, there is extensive work to apply the PILs into the HT‐PEMs, with some representative results summarized in **Table** **1**.^[^ ^16^, ^18^, ^41^, ^42^, ^65^, ^66^, ^67^ ^]^ The results indicate that Nafion/PILs composite membranes have better proton conductivity under high temperature and anhydrous conditions, which is attributed to self‐assembled proton channels by PILs. Despite that, the tensile strength values are generally low. The polymerization of PILs^[^ ^71^ ^]^ or PILs/inorganic fillers co‐doping methods^[^ ^67^ ^]^ have been reported to alleviate the trade‐off and achieved balanced proton conductivity and mechanical strength. For example, Li et al.^[^ ^67^ ^]^ have prepared the Nafion/nano‐SiO_2_/‐OH functionalized PILs composite membranes, where the Nafion and SiO_2_ could be cross‐linked by PILs‐OH through strong hydrogen bonds (Figure 2f, left). The hydrogen‐bond network enabled a synergistic enhancement of mechanical strength and proton conductivity (Figure 2f, middle), finally achieving a maximum power density of 420 mW cm^−2^ at 180 °C and anhydrous conditions (H_2_/O_2_) (Figure 2f, right). Although various strategies have been employed to address the challenges of PFSA‐based composite membranes and achieved balanced proton conductivity and mechanical strength, the actual prospects and feasibility of these strategies still need validation by further investigating the long‐term stability in mechanical properties and conductivity.

**Table 1:** Representative Nafion/ILs composite membranes.

Materials	Chemical structure of ILs	Tension strength [MPa]	Proton conductivity [mS cm^‐1^]	Maximum power density [mW cm^‐2^]
BA‐MS^[^ ^16^ ^]^	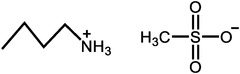	–	0.8 (150 °C, anhydrous)	–
TFTEA^[^ ^18^ ^]^	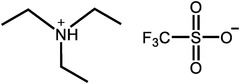	–	6.5(110 °C, anhydrous)	–
[EIm]162^[^ ^41^ ^]^	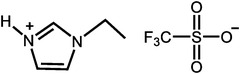	–	1.20(150 °C, anhydrous)	–
[HMIM][FAP]^[^ ^65^ ^]^	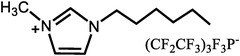	–	1.0(120 °C, anhydrous)	–
[BMIM][BF4]^[^ ^65^ ^]^	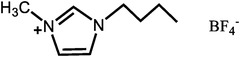	–	0.85(120 °C, anhydrous)	–
[DMBuIm][H2PO4]+GO^[^ ^42^ ^]^	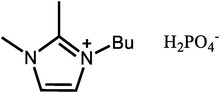	–	61(110 °C, anhydrous)	20(110 °C,H_2_/O_2_)
[MimAE]Cl+PA^[^ ^66^ ^]^	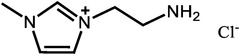	–	6.0(130 °C, anhydrous)	–
[C3OHmim][BF4]+SiO2^[^ ^67^ ^]^	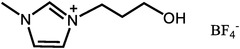	11.5	39(160 °C, anhydrous)	340(160 °C,H_2_/O_2_)

‐ represents that no specific value is given in the references.

#### 2.1.2 SHP‐based Membrane

Sulfonated hydrocarbon polymers (SHPs) possess excellent characteristics, such as good thermal stability, high resistance to chemical oxidation, and high mechanical strength, which are considered as alternatives to replace the PFSAs to be the candidates of HT‐PEMs. To realize the application in HT‐PEMFCs, good proton conductivity is necessary. One of the most efficient approaches is sulfonation, and some representative materials are sulfonated polyether ether ketone (SPEEK), sulfonated poly(arylene ether ketone sulfone) (SPAEKS), sulfonated polyphenylene sulfide (SPPS), sulfonated poly(ether sulfone) (SPES), and sulfonated polyimides (SPI).^[^ ^72^, ^73^, ^74^ ^]^ However, the proton conductivity of many SHP‐based membranes are still very low, especially under high temperature and low humidity condition (<40%RH), compared to that of PFSA‐based membranes.^[^ ^74^ ^]^ To resolve the problem, researchers have introduced hygroscopic materials (e.g. TiO_2_, SiO_2_, GO and its derivatives),^[^ ^75^, ^76^, ^77^ ^]^ or hydrophilic protic carriers (HPA, POSS derivatives, and protic ILs)^[^ ^78^, ^79^, ^80^, ^81^, ^82^ ^]^ into the SHP‐based matrix, and yielded the composite membranes with improved conductivity and comparable mechanical properties. **Table** **2** summarized some of the literature about the SHPs/additives composite membranes.^[^ ^78^, ^79^, ^80^, ^81^, ^82^, ^83^, ^84^, ^85^, ^86^, ^87^, ^88^ ^]^ For example, Kang et al.^[^ ^83^ ^]^ have developed a thermochemically stable and proton conductive sulfonated aromatic PEM based on poly(arylene perfluorophenylsulfonic acid)s (denoted as SP100) and achieved 40 mS cm^−1^ and 232 mS cm^−1^ at 120 °C‐50%RH and 120 °C‐90% RH, respectively. Zhang et al.^[^ ^89^ ^]^ have prepared a SPEEK/HPW composite membrane with improved proton conductivity, and it was comparable to that of Nafion 212 membrane. Although additive engineering has successfully improved the proton conductivity of SHP‐based membranes, the optimal one is only comparable to that of Nafion. Therefore, there is still a long way for SPHs to replace PFSAs.

**Table 2:** Some representative SHPs membranes and corresponding composite membranes.

Composite membranes	Tension strength [MPa]	Proton conductivity [mS cm^−1^]	Maximum power density [mW cm^−2^]
SPEEK^[^ ^78^ ^]^	–	76(100 °C)	–
SP100^[^ ^83^ ^]^	–	140(120 °C, 50%RH)	–
SPIH^[^ ^84^ ^]^	–	1670(120 °C,100%RH)	–
SPAES^[^ ^85^ ^]^	–	200 (140 °C, 100%RH)	–
SPEEK/ZrP‐NS^[^ ^86^ ^]^	–	79(150 °C, 100%RH)	–
SPEEK/TPA^[^ ^78^ ^]^	–	95(100 °C)	–
SPEEK/[BMIm][BF_4_]^[^ ^79^ ^]^	19.8	9.3(140 °C, anhydrous)	–
SPEEK/[BMIm]/PA^[^ ^80^ ^]^	12.8	73.8(160 °C, anhydrous)	–
SPEEK/ SiO_2_/PWA^[^ ^87^ ^]^	–	6.25(100 °C, 90%RH)	–
SPEEK/SiO_2_/[BMIm][BF_4_]^[^ ^88^ ^]^	–	15(200 °C, anhydrous)	–
SPEEK/SiO_2_/[BMIm]^[^ ^88^ ^]^	–	2.9(180 °C, anhydrous)	–
SPEEK/[EMIm][DEP]^[^ ^81^ ^]^	–	1.25–3.16(145 °C, anhydrous)	203(145 °C,H_2_/O_2_)
SPEEK/SBA‐15/Si‐Imi^[^ ^82^ ^]^	23.0	10.2(140 °C, anhydrous)	183(145 °C,H_2_/O_2_)

‐ represents that no specific value is given in the references.

For SA‐containing membranes, the emergence of SSC‐PFSAs has expanded the operating conditions of PFSA‐based membranes, which enabled their application in moderate temperature (100–120 °C) and low‐relative humidity fuel cells. The additive engineering has also enabled the Nafion membranes to work at 100–120 °C, but both the proton conductivity and performance are still low. Combining the advantages of SSC‐PFSAs and additives, we believe the future work should focus on the development of SSC‐PFSA/additive composite membrane, which is expected to be the promising one in the SA‐based HT‐PEMs. Due to the intrinsic physicochemical properties and proton transport mechanism of SA‐containing materials, the optimal working conditions for SA‐based HT‐PEMs are limited at moderate temperatures of 100–120 °C and humidity environment.

### 2.2 Phosphorite Acid (PA)‐Containing Membranes

#### 2.2.1 PA‐PBI Membranes

**Figure 3:**
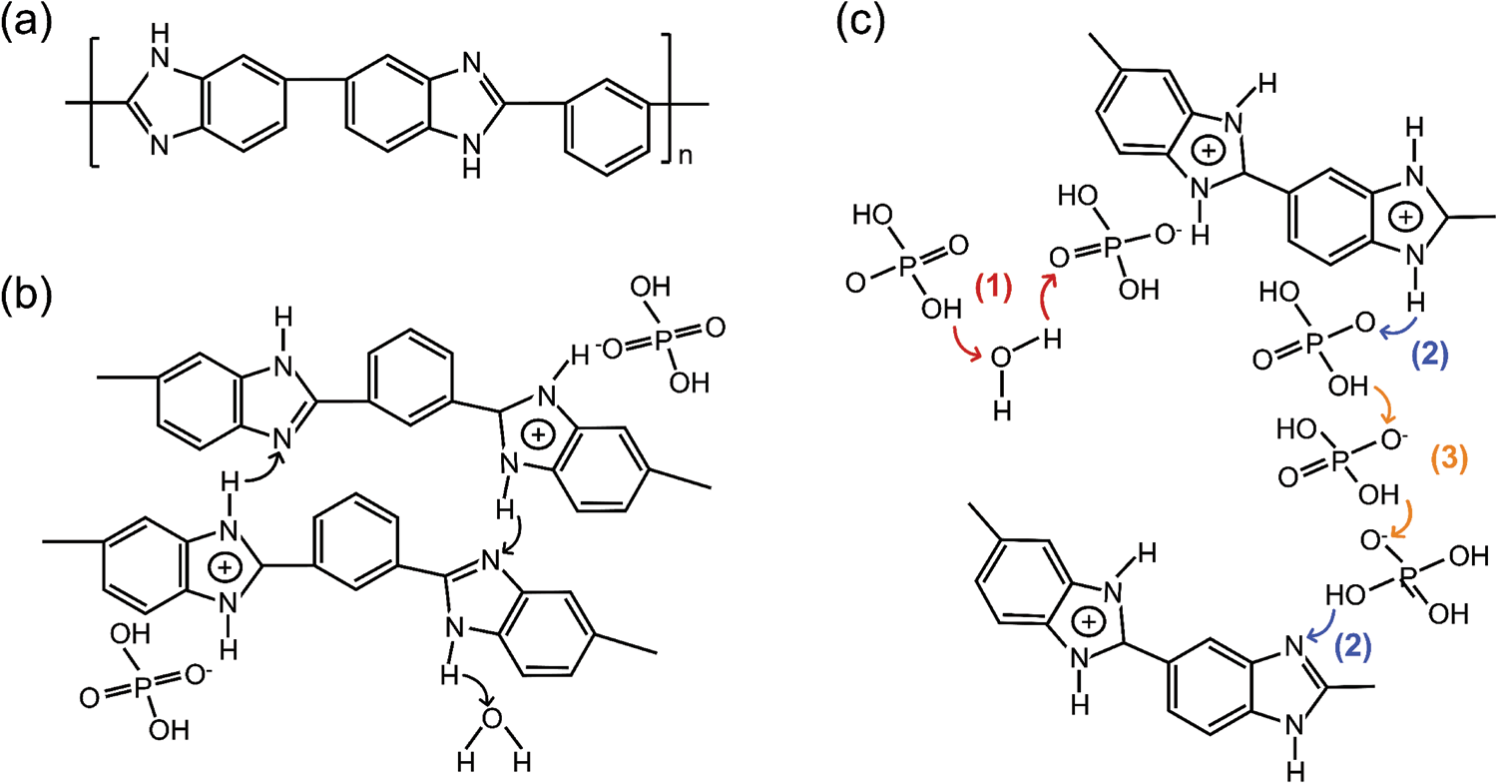
The chemical structures of a) PBI polymer, and b) PA‐PBI. a,b) Reproduced with permission.^[^ ^92^ ^]^ Copyright 2004, Electrochemical Society. c) The proton conductivity mechanism of PA‐PBI membranes includes three possible paths: 1) water‐phosphoric acid proton transfer, 2) benzimidazole ring‐phosphoric acid proton transfer, and 3) proton transfer through phosphoric acid. Reproduced with permission.^[^ ^73^ ^]^ Copyright 2010, Royal Society of Chemistry.

PBI is a class of aromatic heterocyclic polymers containing benzimidazole repeating units, which has acceptable mechanical strength, chemical stability, and thermal stability with a high *T*_g_ of 425–436 ^o^C.^[^ ^90^ ^]^ Among the various PBI, mPBI (poly[2,2’‐m‐(phenylene)‐5,5’‐bibenzimidazole]) has been the most frequently researched material for HT‐PEMFCs, which are more suitable for working at temperatures beyond 140 °C. For pristine PBI membranes, the intrinsic proton conductivity is very low (10^−9^ mS cm^−1^), limiting the direct application in HT‐PEMFCs.^[^ ^91^ ^]^ Acid doping, such as sulfuric acid (H_2_SO_4_), phosphoric acid (H_3_PO_4_), perchloric acid (HCl), and hydrochloric acid (HNO_3_), has been confirmed to be an efficient method to improve proton conductivity.^[^ ^9^ ^]^ Among them, H_3_PO_4_ is the most favorable one due to its excellent proton conductivity at high temperatures and anhydrous conditions. In H_3_PO_4_ doped PBI (PA‐PBI) membranes, the proton conduction is dependent on the Grotthus‐type hopping mechanism, with the possible transfer paths shown in **Figure** **3**.^[^ ^73^, ^92^ ^]^ The proton transfer speed of different paths is H_3_PO_4_(H_2_PO_4_^−^)^…^H─O─H>H_3_PO_4_^…^H_2_PO_4_^−^>N─H^…^ H_2_PO_4_^−^ >N─H^…^H─O─H>N─H^…^N─H, indicating the dominance of H_3_PO_4_ in determining the proton conductivity.^[^ ^92^ ^]^ Previous studies have shown that the proton conductivity increases with the PA doping levels but at the cost of mechanical strength.^[^ ^93^ ^]^ A representative example is that when the doping amount of PA increased from 3.28 to 4.62 mol per repeat unit of PBI, the proton conductivity increased from 20 to 60 mS cm^−1^, while the mechanical strength decreased from 121 to 33 MPa.^[^ ^19^ ^]^ Except for the trade‐off between high proton conductivity and weak mechanical strength induced by the high PA doping levels, PA leaching is another issue that requires attention. At present, different strategies have been explored to mitigate the drawbacks of PA‐PBI membranes, which can be categorized into three parts: 1) alternating the chemical structure of PBI, 2) cross‐linking treatment, and 3) introducing additives. In the following sections, we will discuss this in detail.

##### Chemical Structure Manipulation of PBI

**Figure 4:**
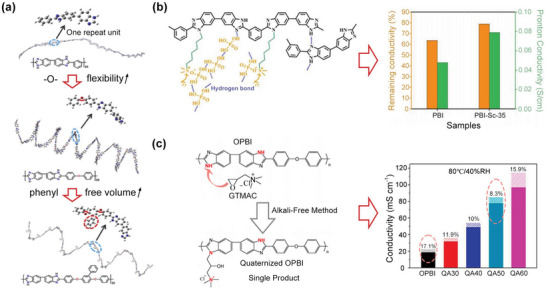
Representative strategies to modify the PBI polymers. a) The molecular simulation results indicate that incorporating ‐O‐ and bulky pendent groups can improve flexibility and free volume. Reproduced with permission.^[^ ^21^ ^]^ Copyright 2016, Elsevier. b) The modification of PBI with quaternized side chains can efficiently improve the proton conductivity and its stability under 150 °C and anhydrous conditions. Reproduced with permission.^[^ ^24^ ^]^ Copyright 2020, Elsevier. c) Left subfigure: synthetic process of quaternized PBI using green and alkali‐free approach, Right subfigure: improved proton conductivity and good PA retention at 80 °C and 40% RH. Reproduced with permission.^[^ ^25^ ^]^ Copyright 2022, Elsevier.

Altering the chemical structure is the most straightforward approach to dealing with the issues in PA‐PBI membranes. In the past two decades, various types of PBI have been developed, with representative examples summarized in **Table** **3**.^[^ ^19^, ^20^, ^21^, ^22^, ^23^, ^24^, ^25^ ^]^ Poly[2,2’‐(p‐phenylene)‐5,5’‐bibenzimidazole] (pPBI) with para‐structure showed excellent tensile strength and high stiffness compared with mPBI. Kim et al.^[^ ^19^ ^]^ have prepared the PA‐pPBI membranes from methanesulfonic acid solution (MSA casting), and the PA doping levels reached 8.05 mol per repeat unit of PBI, delivering a maximum proton conductivity of 141 mS cm^−1^ at 180 °C‐1% RH and a tensile strength of 60 MPa, which was far beyond that of PA‐mPBI membrane with reasonable PA doping levels of 5 mol. Further, by introducing aromatic ether bonds^[^ ^20^ ^]^ or bulky groups^[^ ^21^ ^]^ into the main chain of pPBI, the flexibility and solubility have been improved (**Figure** **4**a), making it easy to prepare high‐quality membranes. For example, Li et al.^[^ ^20^ ^]^ have prepared the PA‐OPBI membranes with significantly improved thermal stability (decomposition temperature above 500 °C), and a high proton conductivity of 83 mS cm^−1^ at 150 °C and anhydrous condition, producing a maximum power density of 1.17 W cm^−2^ at 150 °C with dry gas.

**Table 3:** Chemical structure manipulation of PBI.

Materials	Chemical Structure	Tension strength [MPa]	Proton conductivity [mS cm^‐1^]	Maximum power density [mW cm^‐2^]
pPBI^[^ ^19^ ^]^	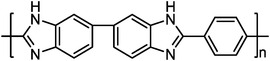	57 (22 °C, 24%RH)	141 (180 °C, 1%RH)	–
OPBI^[^ ^20^ ^]^	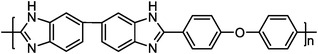	12.9	83 (150 °C)	1170 (150 °C, H_2_/O_2_)
PhPBI/Me‐PBI^[^ ^21^ ^]^	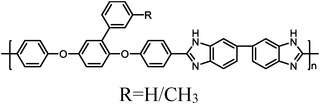 R=H/CH_3_	10.7 (Me‐72)	123 (200 °C, Me‐72)	320 (160 °C, H_2_/air, Me‐72)
F_6_PBI^[^ ^22^ ^]^	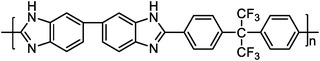	23 (PBI‐3.0PA)	0.17 (160 °C, PBI‐3.0PA)	–
OHPyPBI^[^ ^23^ ^]^	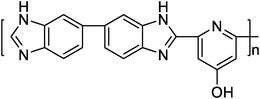	4.6 (OHPyPBI/8.6PA)	102 (180 °C, OHPyPBI/8.6PA)	570 (180 °C, H_2_/O_2_, OHPyPBI/8.6PA)
PBI‐Sc‐X^[^ ^24^ ^]^	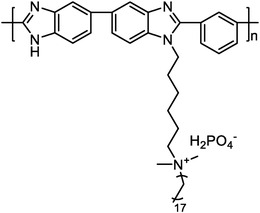	8 (PBI‐Sc‐5) 5 (PBI‐Sc‐35)	104 (170 °C, PBI‐Sc‐35)	411.7 (160 °C, H_2_/air, PBI‐Sc‐5)
quaternized OPBI^[^ ^25^ ^]^	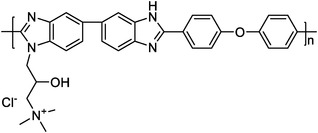	7.1 (QA50)	85 (160 °C, QA50)	355 (160 °C, H_2_/air, QA50)

‐ represents that no specific value is given in the references.

Functionalizing backbones or introducing side chains into the PBI is also an efficient method to improve the physicochemical properties and durability of membranes.^[^ ^23^, ^24^, ^25^ ^]^ Yang et al.^[^ ^23^ ^]^ have prepared the poly[2,2’‐(m‐phenylene)‐5,5’‐bibenzimidazole] (OHPyPBI), and an improved proton conductivity of ≈102 mS cm^−1^ and a maximum power density of 570 mW cm^−2^ at 180 °C were achieved using the PA‐OHPyPBI membranes. The good performance could be attributed to the increased PA doping levels induced by the increased alkaline groups (‐N═ ) and the extra hydrogen bond networks induced by the hydroxyl groups (‐OH). By introducing strong basic groups, such as quaternary ammonium, imidazolium group, and pyridium, onto the backbones or side chains, the PA doping levels and PA retention properties have been improved.^[^ ^25^ ^]^ For example, Chen et al.^[^ ^24^ ^]^ have grafted alkyl chains with quaternary ammonium end group onto PBI to prepare side chain quaternized PBI and the resulting membrane exhibited great PA retention and a high conductivity of 104 mS cm^−1^ at 170 °C (Figure 4b). The results indicated that the electrostatic forces between quaternary ammonium and bisphosphate, and extra hydrogen bridges are the key reasons for the increased PA doping levels, enhanced PA retention, and improved proton conductivity. However, traditional methods for quaternization of PBI are defective due to the usage of toxic halogenated hydrocarbons and alkaline. He et al.^[^ ^25^ ^]^ have developed a green and alkaline‐free method to quaternize the OPBI (QOPBI) (Figure 4c), and the PA‐QOPBI membrane with 50% quaternization degree enables a high PA doping level of 18.8, anhydrous conductivity of 85 mS cm^−1^ at 160 °C, and good operation stability even under 80 °C and 40% RH.

##### Cross‐linked PBI Membrane

**Figure 5:**
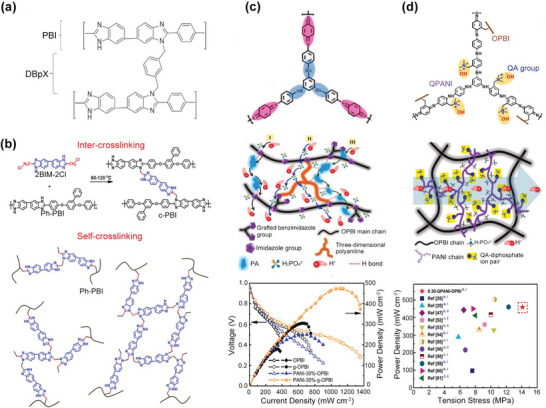
Representative cross‐linkers and corresponding interaction mechanism. a) Representative of small molecule cross‐linking agents‐DBpX. Reproduced with permission.^[^ ^26^ ^]^ Copyright 2017, Elsevier. b) Imidazole‐rich cross‐linker (2BIM‐2Cl), which have inter‐cross‐linking and self‐cross‐linking effects. Reproduced with permission.^[^ ^27^ ^]^ Copyright 2020, American Chemical Society. c) The chemical structure of PANI cross‐linkers, the schematic illustrating the proton conductivity mechanism of PANI/PA‐PBI membranes, and the polarization performance under 160 °C. Reproduced with permission.^[^ ^29^ ^]^ Copyright 2022, Elsevier. d) The chemical structure of quaternized PANI (QPANI) cross‐linkers, the schematic illustrating the proton conductivity mechanism of QPANI/PA‐PBI membranes, and the polarization performance under 160 °C. Reproduced with permission.^[^ ^30^ ^]^ Copyright 2022, Elsevier.

Cross‐linking, especially covalent cross‐linking, has been found to be a good strategy to improve the overall characteristics, including chemical stability, PA retention, and mechanical strength. In previous studies, small molecule cross‐linkers, such as terephthalaldehyde (TPA), bisphenol A diglycidyl ether (BADGE), ethylene glycol diglycidyl ether (EGDE), and α‐α’‐dibromo‐p‐xylene (DBpX), have been used to cross‐link the PBI polymers by forming a covalent bond with imidazole ring (**Figure** **5**a),^[^ ^26^ ^]^ and succeeded in improving the mechanical strength and PA retention capacity. However, the direct interaction is at the expense of free imidazole groups in PBI, thus reducing the acid doping ability and proton conductivity. In order to address the trade‐off between strength and conductivity, researchers have developed various cross‐linkers with different functional groups, such as tetrafunctional bischloro/bibenzimidazole (2BIM‐2Cl),^[^ ^27^ ^]^ brominated gamma‐(2,3‐epoxypropxy)propyltrimethoxysilane (KH560),^[^ ^28^ ^]^ polyaniline (PANI) derivatives,^[^ ^29^, ^30^ ^]^ and bishydroxymethyl‐type cross‐linker(2,6‐bis(hydroxymethyl)‐4‐methylphenol (BHMP),^[^ ^31^ ^]^ which can not only cross‐link the PBI polymers, but also provide extra active sites to capture the PA molecules. For example, Li et al.^[^ ^27^ ^]^ have prepared the 2,2′‐Bis(chloromethyl)‐5,5′‐Bibenzimidazole (2BIM‐2Cl) cross‐linked PBI membranes, in which the inter‐cross‐linking of PBI and 2BIM‐2Cl and self‐cross‐linking of 2BIM‐2Cl were coexisting (Figure 5b). The self‐cross‐linking effect enabled a large free volume and imidazole group‐rich regions, which ensured a higher PA doping level, a better proton conductivity, and a maximum power density of 533 mW cm^−2^ at 160°C and anhydrous. The PANI and its derivatives are another class of cross‐linkers, and the specific three‐dimensional structure and ‐NH groups‐containing could efficiently balance the proton conductivity and mechanical properties (Figure 5c). Based on the PANI/OPBI membrane and PANI/g‐OPBI membranes, the single cell performances are significantly improved, which are 250 mW cm^−2^ and 447 mW cm^−2^ at 160 °C and anhydrous, respectively.^[^ ^29^ ^]^ The functionalization of PANI using quaternary ammonium (QA) groups further enhanced the PA uptake ability, thus allowing for further improvement of proton conductivity (145.6 mS cm^−1^) and peak power density (459 mW cm^−2^) at 160 °C and anhydrous condition (Figure 5d).^[^ ^30^ ^]^

##### PA‐PBI Composite Membrane

Introducing inorganic or organic fillers into the PA/PBI matrix to prepare composite membranes is an effective way to improve the performance and durability of HT‐PEMFCs. **Table** **4** provided a summary of some representative PA/PBI composite membranes and the corresponding key performance parameters.^[^ ^32^, ^33^, ^34^, ^35^, ^36^, ^94^, ^95^, ^96^, ^97^, ^98^, ^99^, ^100^ ^]^ Although the introduction of pristine SiO_2_, TiO_2_, graphene oxide (GO), and other oxides have successfully improved the PA holding capacity and prevented acid leaching by the hydrogen‐bonding effect, the weak compatibility between PBI and oxides led to the poor dispersion properties, thus making it difficult to obtain repeatable results and to industrialization. Moreover, the absence or limited proton transfer sites also limited the further improvement of performance. The functionalization of oxides has been confirmed to be an efficient method to address the low proton conduction issue.^[^ ^94^, ^95^, ^96^, ^97^, ^98^ ^]^ For example, grafting the 3‐aminopropyltriethoxysilane ionic liquid,^[^ ^94^ ^]^ 3‐amino‐1,2,4‐triazole (Am‐Tri),^[^ ^95^ ^]^ and sulfonic acid group^[^ ^96^ ^]^ on the surface of GO have successfully improved the comprehensive properties of composite membranes, such as reinforced PBI matrix, improved dispersion uniformity, and improved proton conductivity. Yang et al.^[^ ^95^ ^]^ have prepared the triazole‐modified graphene oxide (MGO)/PBI composite membrane, which delivered a high proton conductivity of 0.135 S cm^−1^ at 180 °C and an excellent peak power density of 537 mW cm^−2^.

**Table 4:** Key performance parameters of some representative PA/PBI composite membranes.

Composite membranes	PA uptake [%/ADL]	Tension strength [MPa]	Proton conductivity [mS cm^−1^]	Maximum power density [mW cm^−2^]
ILGO/PBI 3.5^[^ ^94^ ^]^	‐/3.5	–	35 (175 °C)	320 (175 °C, H_2_/O_2_)
PBI/MGO/12.2PA^[^ ^95^ ^]^	‐/12.2	12.6	135 (180 °C)	537 (180 °C, H_2_/O_2_)
SGO/PBI/PA^[^ ^96^ ^]^	‐/1.9	–	52 (175 °C)	600 (175 °C, H_2_/O_2_)
OPBI/LAMS‐10%^[^ ^97^ ^]^	‐/24.3	–	176 (160 °C)	–
OPBI/ILMS‐15%^[^ ^98^ ^]^	‐/36.5	–	240 (160 °C)	–
ABPBI/2S‐Sep^[^ ^32^ ^]^	–	–	51 (180 °C, ABPBI/2S‐Sep‐1.92PA)	230 (180, H_2_/O_2_, ABPBI/2S‐Sep‐1.85PA)
ABPBI/5PEI@SNR^[^ ^99^ ^]^	–	54	40 (180 °C)	270 (180 °C, H_2_/O_2_)
30%‐CTFs‐OPBI^[^ ^100^ ^]^	167.1/–	7.7	71.7 (160 °C)	534.4 (160 °C, H_2_/O_2_)
40%–COF–OPBI^[^ ^33^ ^]^	387.3/–	6.0	177.7 (160 °C)	774.7 (160 °C, H_2_/O_2_)
40%UIO‐66@OPBI^[^ ^34^ ^]^	73.25/–	27.02	92 (160 °C)	583 (160 °C, H_2_/O_2_)
10%‐ILs/NH_2_‐CNTs/OPBI^[^ ^35^ ^]^	175.9/8.3	16.3	130.8 (160 °C)	508 (160 °C, H_2_/O_2_)
CBOPBI@MOF50%‐IL30^[^ ^36^ ^]^	93/–	–	135 (160 °C)	736 (160 °C, H_2_/O_2_)

‐ represents that no specific value is given in the references.

HPA is a good inorganic proton conductor without any functionalization treatment, making it a good additive in PA‐PBI membranes. However, the good solubility in water made it prone to loss during fuel cell operation. Therefore, various strategies have been conducted to stabilize the HPAs. For example, Staiti et al.^[^ ^101^ ^]^ have developed prepared SiO_2_/HPA composite nanoparticles, in which the HPA is stabilized by the SiO_2_ porous structure. When the SiO_2_/HPA nanoparticles were incorporated into the PBI matrix, a maximum power density of 660 mW cm^−2^ was achieved at 120 °C and 40% RH. Besides, other porous materials, such as MOF and COF, have also successfully stabilized the HPAs.^[^ ^14^ ^]^ Except for acting as the stabilizer, the functionalized COF and MOF have also been widely incorporated into the PBI matrix to prepare high‐temperature composite membranes.^[^ ^33^, ^34^ ^]^ For instance, Peng et al.^[^ ^33^ ^]^ have fabricated a polyarylether functionalized COF‐OPBI composite membrane, which showed excellent overall performances, such as high proton conductivity (177 mS cm^−1^), desirable mechanical tensile strength (12.1 MPa), and high power density (774.7 mW cm^−1^) at 160 °C without humidification.

Protic ionic liquid (PIL) is a type of room‐temperature molten salt with high thermal stability and good proton conductivity, which is the potential material for HT‐PEMs.^[^ ^35^, ^36^ ^]^ Incorporating PIL into PBI matrix can reduce the dependence of proton conductivity on PA doping amount, thus weakening the problems of poor mechanical properties and serious PA loss caused by high doping levels. However, a single PIL additive faced the risk of losing from membranes or weakening the tension strength of composite membranes. Therefore, it is suggested to use PILs together with other additives, and there are already some successful cases. For example, Xiao et al.^[^ ^35^ ^]^ have prepared the PILs/NH_2_‐CNTs/OPBI hybrid membranes by combining the PILs and carbon nanotubes (CNTs), which possessed superior proton conductivity and excellent mechanical properties. The 10%‐PILs/NH_2_‐CNTs/OPBI membrane exhibited a better tensile strength of 18.1 MPa after PA doping and better ILs retention ability compared with 10%‐ILs/OPBI membrane.

The previous research indicated that the optimal operation conditions of PA‐PBI‐based HT‐PEMFCs are at 140–200 °C and an anhydrous environment. The modification of PA‐PBI membranes by chemical structure manipulation, cross‐linking treatment, or introducing some protic carriers has successfully improved the mechanical strength, proton conductivity, or PA retention. Despite that, the modified PA‐PBI membranes still face serious PA loss under high humidity conditions and suffer from low proton conductivity at low temperatures, thus limiting the practical application. Therefore, further investigations are still needed for the PA‐PBI membranes to broaden the working temperature and humidity window, and reduce the temperature and humidity sensitivity.

#### 2.2.2 PA‐Polymer Containing Basic Groups

**Figure 6:**
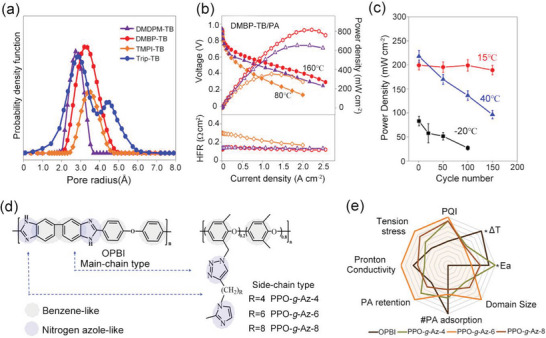
a) The pore size distribution of TB‐based polymers, which were obtained via CONTIN analysis from PALS. b) The H_2_/O_2_ polarization performances of PA‐doped DMBP‐TB membranes under 80 and 160 °C. c) The peak power density of the DMBP‐TB/PA membranes after the shut‐down/start‐up AST cycling at −20 °C, 15 °C, and 40 °C. The polarization curves of the shut‐down and start‐up AST cycling tests for PA doped DMBP‐TB membranes. a–c) Reproduced with permission.^[^ ^38^ ^]^ Copyright 2022, Springer Nature. d) The chemical structure of main‐chain‐type OPBI and side‐chain‐functionalized PPO‐g‐Az‐6. e) The PPO‐g‐Az‐6 membranes with balanced and impressive properties. d,e) Reproduced with permission.^[^ ^39^ ^]^ Copyright 2023, Wiley‐VCH.

Polymers containing strong basic groups are good candidates for replacing the PBI‐based polymers, with some representative examples summarized in **Table** **5**.^[^ ^37^, ^38^, ^39^, ^102^, ^103^, ^104^, ^105^, ^106^, ^107^ ^]^ The strong basic sites in backbones or side chains could efficiently rivet the PA molecules, thus enabling its possible application in wide temperature and humidity windows. For example, Lee et al.^[^ ^37^ ^]^ have prepared quaternary ammonium (QA)‐biphosphate ion‐pair‐coordinated polyphenylene (PA‐doped QAPOH) PEMs, and the strong QA^+^…H_2_PO_4_^−^ interactions enabled a high PA retention, a stable conductivity, and improved performance at 80–160 °C. Tang et al^[^ ^38^ ^]^ have prepared four Tröger's base (TB)‐based polymers, and through phosphorylation treatment, four HT‐PEMs with ultra‐micropore radium were obtained (**Figure** **6**a). Both the siphoning effect of microporosity and the strong acid‐base interactions allowed high PA retentions even under high humidity conditions and enabled stable operation from ‐20 to 200 °C (Figure 6b,c). The DMBP‐TB/PA membrane exhibited good device performances under each operating condition (815 mW cm^−2^ @ 160 °C and 83 mW cm^−2^ @ ‐20 °C under H_2_/O_2_ condition). After 150 cycles of start‐up/shut‐down testing, the DMBO‐TB/PA membrane displayed 95% power density retention at 15 °C and can accomplish over 100 cycles even at −20 °C (Figure 6c). Besides, Zhang et al.^[^ ^39^ ^]^ have developed novel a material (pendent imidazole‐functionalized polyphenylene oxide (PPO)) with the PA molecules trapping sites located in the side chains. The design rules can not only weak plasticizing effect caused by PA toward the polymer backbone to maintain good tensile stress, but also induce micro‐phase separation structure to improve the PA retention (Figure 6d,e). The results showed that PA/PPO‐g‐Az‐6 membranes survived over 7 times longer than the PA/OPBI membranes under harsh conditions (80 °C‐100% RH). Importantly, it achieved stable operations at wide temperature range from 80 to 160 °C, with a maximum power density of 576 mW cm^−2^ at 160 °C and 190 mW cm^−2^ at 80 °C‐100% RH under H_2_/Air condition. The above successful cases indicate that the future goals of PA‐based membranes should focus on the design of microporous structures and their functionalization.

**Table 5:** Key performance parameters of some non‐PBI‐based HT‐PEMs reported recently.

Membranes	Chemical Structure	PA uptake [%]	Tension strength [MPa]	Proton conductivity [mS cm^−1^]	Maximum power density [mW cm^−2^]
PA‐doped QAPOH^[^ ^37^ ^]^	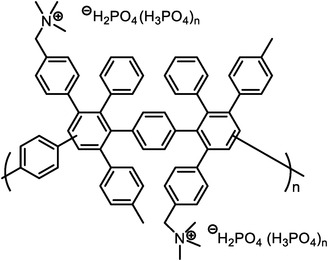	–	–	–	800 (180 °C, H_2_/O_2_)
DMBP‐TB/PA^[^ ^38^ ^]^	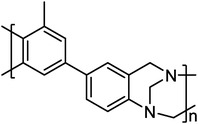	425	–	159 (180°C)	815 (160 °C, H_2_/O_2_)
PA‐doped PPO‐*g*‐Az‐6^[^ ^39^ ^]^	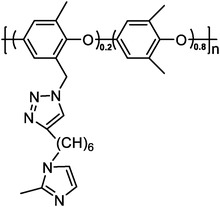	–	12.1	62 (160°C)	576 (160 °C, H_2_/O_2_)
SPFTP‐50^[^ ^102^ ^]^	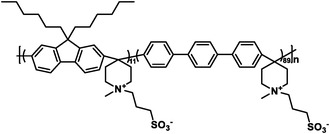	162.8	4.1	–	280 (100 °C, H_2_/air)
PPT/PA^[^ ^103^ ^]^	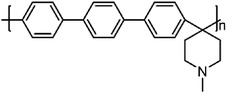	159.93	12	96 (180°C)	1220.2 (180 °C, H_2_/O_2_)
PA doped PPy^104^ ^]^	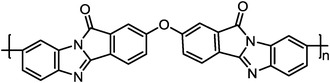	240	–	68 (180°C)	582 (160 °C, H_2_/O_2_)
PTP‐41%BeIm/215%PA^[^ ^105^ ^]^	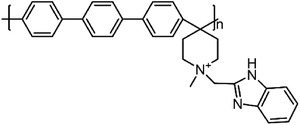	215	3.3	88 (180°C)	995 (180 °C, H_2_/O_2_)
P‐g‐V‐3.82/PA^[^ ^106^ ^]^	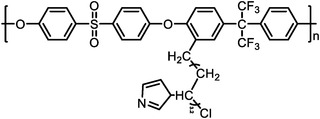	220.3	7.94	127 (160°C)	559 (160 °C, H_2_/O_2_)
PA doped TDAP‐PSU‐88^[^ ^107^ ^]^	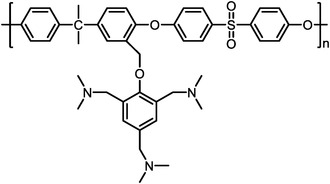	186.35	1.8	46 (160°C)	498 (180 °C, H_2_/O_2_)

‐ represents that no specific value is given in the references.

## 3 Durability of HT‐PEMs

### 3.1 Degradation Mechanisms of SA‐Containing Membranes and Mitigating Strategies

**Figure 7:**
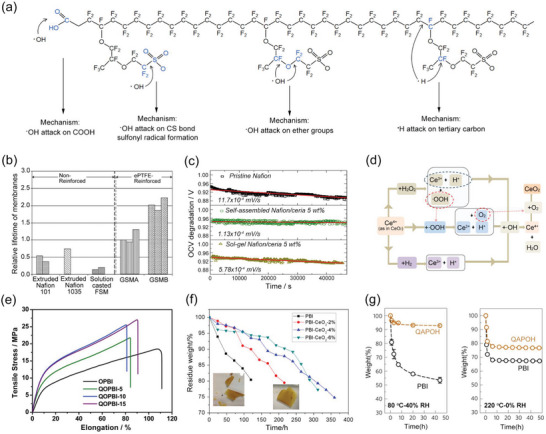
a) The mechanism of chemical degradation for PFSA‐based membranes. Reproduced with permission.^[^ ^112^ ^]^ Copyright 2020, Elsevier. b) Improved durability of ePTFE‐reinforced PFSA‐based membranes. Reproduced with permission.^[^ ^108^ ^]^ Copyright 2008, Elsevier. c) OCV degradation curves of pristine Nafion, self‐assembled Nafion/CeO_2_, and sol‐gel Nafion/CeO_2_. Reproduced with permission.^[^ ^119^ ^]^ Copyright 2012, Elsevier. d) The mechanism of CeO_2_ to quench the radicals. Reproduced with permission.^[^ ^122^ ^]^ Copyright 2012, National Academy of Sciences. e) Mechanical properties of PA‐OPBI membranes without and with hyperbranched cross‐linkers. Reproduced with permission.^[^ ^129^ ^]^ Copyright 2020, Elsevier. f) Accelerated oxidation testing of PBI and PBI/CeO_2_ composite membranes. Reproduced with permission.^[^ ^118^ ^]^ Copyright 2017, Elsevier. g) The weight change percentage of PA‐doped polymers as a function of time at 80 °C‐40% RH and 220 °C‐0% RH, which can be used to assess PA retention. Reproduced with permission.^[^ ^37^ ^]^ Copyright 2016, Springer Nature.

Currently, extensive work has been conducted to reveal the degradation mechanisms of SA‐containing membranes, mostly focusing on PFSA‐based membranes. It is generally accepted that the degradation of PFSA‐based membranes is mainly attributed to three aspects: mechanical stress, thermal decomposition, and chemical oxidation.^[^ ^108^ ^]^ During the assembly process of PEMFCs, the membranes in contact with the flow fields and sealing gaskets were subjected to excessive or non‐uniform mechanical stresses, making them vulnerable to perforations or tears. Additionally, during the operation process of PEMFCs, the change in temperature and humidity led to repeated swelling/shrinkage, further resulting in creep, microcracks, and pinholes in the membranes. R. M. H. Khorasany et al.^[^ ^109^ ^]^ have conducted the fatigue life testing of PEMs under humidity and temperature cycling and pointed out that humidity variation was the key factor in leading to mechanical attenuation of PEMs. For thermal degradation, the thermogravimetric analysis (TGA) of PFSA membranes showed that there was no significant weight loss in temperatures below 300 °C. Therefore, under the typical operating temperature range of HT‐PEMFCs, the PFSA membranes would not experience significant thermal degradation.^[^ ^46^, ^110^ ^]^ For chemical degradation, it generally comes from two aspects:^[^ ^111^ ^]^ 1) the fuel crossover results in the direct reaction between H_2_ and O_2_, and then produces hydroxyl (HO·) and hydroperoxyl (HOO·) radicals, 2) the two‐electron reaction of ORR in the cathode catalyst layers can also lead to the formation of HO· and HOO· radicals. These radicals will attack the vulnerable bonds (C─S, C─O, and C─F bond) in the PFSA structures (**Figure** **7**a), resulting in the loss of membrane integrity and thickness thinning.^[^ ^112^ ^]^ Further investigations have shown that the generation of radicals and chemical degradation rate is accelerated when the PEMFCs are operated under open circuit voltage (OCV) and low humidity conditions.^[^ ^113^ ^]^ In addition, some metal ions (such as Fe^2+^, Cu^2+^) coming from the corrosion of end plates or metal bipolar plates can promote the generation of HO· and HOO· radicals, further exacerbating the chemical degradation.^[^ ^93^, ^112^ ^]^

To improve the durability of PFSA‐based membranes, various strategies have been conducted. The most effective method to improve the mechanical strength is to incorporate a thin microporous polytetrafluoroethylene (PTFE) layer to fabricate the composite membranes.^[^ ^114^ ^]^ Gore Fuel Cell Technologies have developed a reinforced membrane with e‐PTFE, which exhibits a lifetime of an order of magnitude longer than that of a non‐reinforced membrane (Figure 7b).^[^ ^108^ ^]^ Similar results were seen in reinforced Aciplex membranes^[^ ^115^ ^]^ and Nafion‐Teflon‐HPW composite membranes.^[^ ^116^ ^]^ With respect to chemical degradation, the effective solution is to introduce the free radical quenching agents into the membranes, such as metal oxides (e.g. CeO_2_, MnO_2_, ZrO_2_)^[^ ^117^, ^118^, ^119^ ^]^ and organic antioxidants (phthalate, 2,6‐Dimethoxy‐1,4‐benzoquinone, 3,4‐dihydroxycinnamic acid).^[^ ^120^, ^121^ ^]^ For example, Zhao et al^[^ ^119^ ^]^ have prepared Nafion/CeO_2_ composite membranes using sol‐gel and self‐assembly, respectively. The slow OCV degradation rates indicate that CeO_2_ can protect PEMs from chemical oxidation, especially the self‐assembled Nafion/CeO_2_ membranes (Figure 7c). Figure 7d showed the radical scavenging mechanism of CeO_2_.^[^ ^122^ ^]^ The Ce^4+^ can oxide the H_2_O_2_, HOO·, and H_2_, and the generated Ce^3+^ can further redox the HO· to form Ce^4+^, which not only constructs the regenerative cycle but also efficiently remove the possible radicals.

### 3.2 Degradation Mechanisms of PA‐Containing Membranes and Mitigating Strategies

The degradation of PA‐containing membranes is mainly caused by mechanical stress, chemical oxidation, and PA leaching. During stacking and the fuel cell operation process, the non‐uniform mechanical stress was applied to the membranes and led to time‐dependent deformation, cracking, and pinholes.^[^ ^93^ ^]^ The membrane defects induced by mechanical degradation would result in serious fuel crossover, and generate more hydrogen peroxide (H_2_O_2_) and radicals (HO· and HOO·). These byproducts would attack the C─H bonds or H‐containing terminated groups (e.g. N─H bonds in the imidazole rings) and thus resulted in the degradation of membranes.^[^ ^93^ ^]^ Li et al.^[^ ^123^ ^]^ have found that the weight loss of PA‐PBI membranes was 15% after exposing them to 3% H_2_O_2_ Fenton reagents at 68 °C for 20 h, which is higher than that of Nafion 117 membrane (1%). The results indicated that PA‐PBI membranes are more vulnerable to chemical oxidation. In addition, PA leaching is also an important factor causing degradation. According to previous work, there are two main reasons for the PA loss:^[^ ^124^, ^125^ ^]^ 1) The hot‐pressing process causes the PA molecules to be pressed out of the membranes during the fabrication of the membrane‐electrode assembly. 2) The free PA molecules can be washed out of the membranes by water. Lee et al.^[^ ^126^ ^]^ have studied energetics between PA‐benzimidazole acid‐base and biphosphate‐ammonium ion pairs using the density functional theory calculations and then proposed a novel PA loss mechanism when exposed to liquid water. The results indicated that the limited ability of PBI matrix to accommodate the PA and water molecules led to the PA loss. The strong interaction in the biphosphate‐ammonium ion pair shifted the equilibrium PA composition to higher values, thus minimizing the PA loss in the presence of water.

Regarding the above degradation factors, the researchers have proposed various mitigating strategies. For PA‐containing membranes, the interactions between polymers contributed to the mechanical properties. With the increase of PA doping levels, these interactions can be disrupted by free PA molecules, resulting in a dramatic decrease in mechanical strength, especially at high temperatures.^[^ ^127^ ^]^ Therefore, the PA doping levels should be carefully controlled to balance the trade‐off between proton conductivity and mechanical strength. Introducing cross‐linkers into the PA‐containing membranes can improve mechanical strength.^[^ ^27^, ^29^, ^30^, ^128^ ^]^ There are generally two types of cross‐linkers: one is that it can cross‐link the polymers but at the expense of imidazole groups of polymers, which leads to enhanced mechanical strength but decreased proton conductivity.^[^ ^26^ ^]^ Another is that it can not only cross‐link the polymers but also increase the acid‐uptaking sites, which can simultaneously improve mechanical strength and proton conductivity.^[^ ^27^, ^28^, ^29^, ^30^ ^]^ For example, Hu et al.^[^ ^129^ ^]^ have incorporated the hyperbranched and quaternary ammonium‐containing cross‐linkers into the PA‐OPBI membranes, which not only cross‐linked the OPBI polymers but also increased the proton transport paths, thus delivering enhanced mechanical strength above 20 MPa (Figure 7e), increased proton conductivity, and improved chemically oxidative resistance. In addition, physical blending with some stable polymers is also an efficient method to increase membrane strength.^[^ ^130^, ^131^, ^132^ ^]^ PTFE is a well‐known polymer with excellent oxidative stability and good mechanical strength. To improve the membrane strength without sacrificing the proton conductivity, it is better to use porous PTFE as the supporting matrix. For example, Cao et al.^[^ ^132^ ^]^ have prepared the porous PTFE‐reinforced dimethylhexadecylamine partially quaternized poly(vinyl benzyl chloride) (qPVBzCl) membrane with a tensile strength of 56.23 MPa, which was significantly higher than that of pristine membrane (9.55 MPa). The above results indicated that introducing cross‐linkers and stable polymers into the membranes not only improved the mechanical strength but also increased the resistance to radicals and improved the chemical stability.^[^ ^28^, ^29^, ^30^, ^31^ ^]^ Moreover, Hao et al.^[^ ^118^ ^]^ have introduced CeO_2_ into the PA‐PBI membranes, and the minor weight loss and good membrane integrity after the Fenton test indicated that the free radical quenchers CeO_2_ can significantly enhance the resistance to chemical oxidation (Figure 7f). To address the issue of PA leaching, the key strategy is to enhance the interactions between PA molecules and membrane materials. Functionalizing the PBI polymers with strong basic groups and developing new polymers with strong basic groups have been confirmed to be efficient in anchoring PA molecules and avoiding PA loss.^[^ ^37^, ^38^, ^103^ ^]^ For example, Lee et al.^[^ ^37^ ^]^ have developed the quaternary ammonium (QA)‐biphosphate ion‐pair‐coordinated polyphenylene (PA‐doped QAPOH) membranes, the strong QA^+^…H_2_PO_4_^−^ interactions maintained higher PA contents in both liquid water (80 °C‐40% RH) and water vapor conditions (200 °C‐0% RH) (Figure 7g). Further, Tang et al.^[^ ^38^ ^]^ have developed polymers with Troger's base, and the intrinsic microporosity of these polymers led to the formation of membranes with ultramicroporous structures. The siphoning effect of microporosity and the strong acid‐base interactions allowed high PA retentions even under highly humid conditions. Therefore, except for the strong basic groups, the microporous structures of membranes are of equal importance in addressing the PA leaching problems.

## 4 Development of HT‐PEMs Characterization Technology

It is well known that morphology has a significant effect on the PEMs’ performance and durability, and plenty of work has been carried out to characterize the morphology and further reveal the “morphology‐property” relationships.^[^ ^133^, ^134^ ^]^ Currently, the most common and effective morphology characterization methods consist of wide‐angle X‐ray scattering (WAXS), transmission electron microscopy (TEM), small‐angle X‐ray scattering (SAXS), resonant X‐ray scattering (RXS), and in situ WAXS and SAXS.^[^ ^135^ ^]^ Combining the scattering and microscopic techniques, the crystallization morphology, the phase separation structure, and morphology evolution process from solution to membrane can be clearly elucidated.

### 4.1 Crystallization Morphology

**Figure 8:**
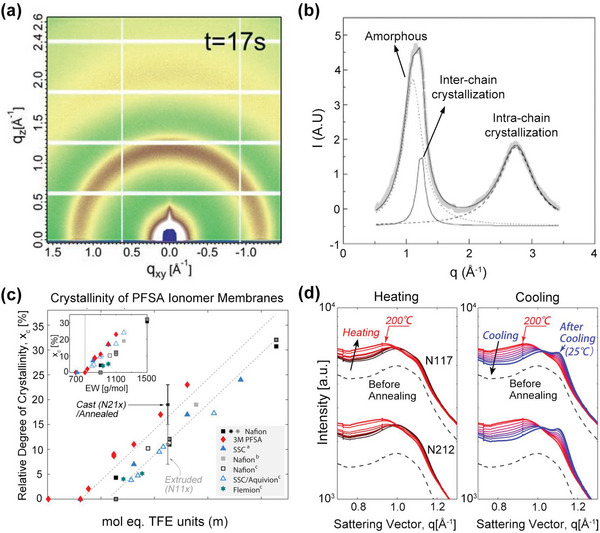
Crystallization morphology of PEMs characterized by WAXS. a) 2D WAXS images of SSC‐PFSA membranes. Reproduced with permission.^[^ ^12^ ^]^ Copyright 2023, American Association for the Advancement of Science. b) WAXS profiles of Nafion 117 with the gray dots representing experimental data, the gray dotted line representing the amorphous peak, the gray solid line representing the interchain crystallization peak, and the black dash line representing intrachain crystallization peak. Reproduced with permission.^[^ ^142^ ^]^ Copyright 2004, American Chemical Society. c) Relative degree of crystallinity for various PFSA membranes plotted as a function of backbone length (TFE units). Reproduced with permission.^[^ ^133^ ^]^ Copyright 2017, American Chemical Society. d) WAXS profiles for Nafion 117 and 212 membranes during in situ heating and cooling process. Reproduced with permission.^[^ ^144^ ^]^ Copyright 2012, American Chemical Society.

In PEMs, crystallization plays an important role in determining PEMs properties, such as mechanical and thermal stability,^[^ ^136^, ^137^ ^]^ gas permeability,^[^ ^138^ ^]^ water uptake, and swelling.^[^ ^139^, ^140^, ^141^ ^]^ The high crystallinity tends to enhance durability but limits proton transport. Therefore, it is significant to carefully manipulate the crystalline properties of PEMs to achieve the balance between durability and proton transport ability. Commonly, the crystallization of PEMs is characterized by wide‐angle X‐ray scattering (WAXS) or traditional X‐ray scattering (XRD). Compared with the traditional XRD, the high energy X‐rays sources and area detector make the WAXS an efficient method to characterize the weak crystalline polymers and make sure the comprehensive collection of signals. **Figure** **8**a showed the 2D WAXS images of SSC‐PFSA membranes. The scattering rings indicated the isotropic crystal orientation of PFSA materials.^[^ ^12^ ^]^ Figure 8b showed the WAXS profile of Nafion 117 membrane, with the fitting curves shown in the inset.^[^ ^142^ ^]^ The peak at ≈1.1 Å^−1^ was assigned to the amorphous phase, the peak at ≈1.2 Å^−1^ was attributed to the backbone crystallization of PFSA, and the peak at ≈2.75 Å^−1^ represented the intrachain crystallization. By fitting each characteristic peak, the quantitative crystallization parameters, such as peak intensity, the crystal coherence length (CCL), and backbone packing layers can be obtained. The degree of the crystallinity (*x_c_*) can be calculated using the following equation,^[^ ^143^ ^]^

(1) $$x_{c}=\frac{\int q^{2}I_{c}\left(q\right)dq}{\int (q^{2}I_{c}\left(q\right)+q^{2}I_{a}\left(q\right))dq}$$

Here, *q* is the scattering vector, *I*_c_(*q*) is the intensity of crystallization peak, and *I_a_* (*q*) is the intensity of amorphous peak. Figure 8c summarized the relative degree of crystallinity for a series of PFSA membranes.^[^ ^133^ ^]^ The results indicated that the crystallinity increased with the backbone length (TFE units) and equivalent weight (EW) increasing, but was also affected by the side chains. At the same EW, PFSA with a short side chain has higher crystallinity. Besides the PFSA chemistry, the influence of external factors, such as annealing treatment^[^ ^144^, ^145^ ^]^ and mechanical stressing,^[^ ^142^, ^143^, ^144^ ^]^ on the crystalline properties of PEMs have also been extensively studied by this technique. In a rather wide range of temperature‐dependent WAXS studies, the crystallinity can be enhanced by heating the PEMs above its glass transition temperature (*T*_g_, e.g. 130–250 °C). Figure 8d provided the WAXS profiles for Nafion 117 and 212 membranes during in situ heating and subsequent cooling process.^[^ ^144^ ^]^ When cooling the membranes from 200 °C to 25 °C, the crystallization peak appeared and then gradually increased, indicating the improved crystallinity after thermal treatment, which could explain how annealing influences the membrane's mechanical properties and water‐uptake properties.

### 4.2 Phase Separation Structure

The phase separation structure of PEMs is one of the most important features governing the PEMs' properties.^[^ ^133^, ^146^ ^]^ A well‐defined microphase separation with continuous ionic clusters evenly dispersed in the semi‐crystalline fluorocarbon backbone matrix is considered as the most efficient structure, enabling efficient proton transport and high durability. Various methods are utilized to reveal the phase separation structure, which includes TEM, and SAXS/RXS.^[^ ^146^, ^147^, ^148^, ^149^ ^]^ TEM can provide direct morphological picture, but in quite localized areas. Scattering techniques provide good size statistics and probe relatively larger areas, thus providing a more homogenized morphology. The scattering signals of SAXS are typically related to the electron‐density difference of each component, and sometimes the limited electron‐density differences make the scattering signals obscure and data analysis challenging. The chemically sensitive properties of RXS make it an efficient method to probe the phase separation by utilizing the difference of feature elements in fluorocarbon backbones and side chains.

#### 4.2.1 Transmission Microscopy Methods

**Figure 9:**
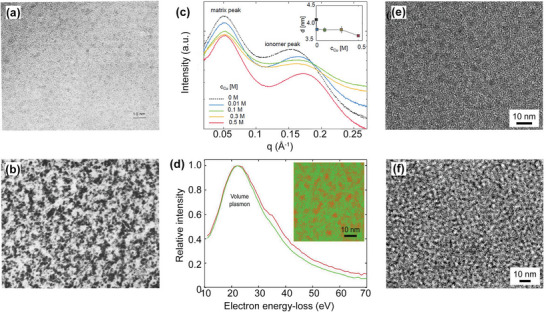
Phase separation structures of PEMs characterized by TEM. TEM images of dry Nafion membranes a) without and b) with RuO_4_ stain. a,b) Reproduced with permission.^[^ ^153^ ^]^ Copyright 1989, Elsevier. c) SAXS profiles of Nafion membranes immersed in Cu solutions of different concentrations, and inset shows the spacing of hydrophilic clusters. d) EF‐TEM chemical mapping for the ionic clusters (red) and hydrophobic matrix (green) and e) bright‐filed TEM of a dry 100 nm Nafion membrane, f) Bright‐filed cryo‐TEM of a frozen‐hydrated 100 nm Nafion membrane. c–f) Reproduced with permission.^[^ ^156^ ^]^ Copyright 2014, American Chemical Society.

Transmission microscopy (TEM) is used extensively as an in‐house technique that is highly accessible and is proven to be an effective method to obtain the direct phase separation picture.^[^ ^150^, ^151^, ^152^ ^]^ Different operational modes in TEM lead to quite varied microscopy techniques, such as bright‐field TEM, high‐angle annular dark‐field (HAADF), scanning transmission electron microscopy (STEM), and energy‐filtered TEM (EF‐TEM). Earlier TEM studies on the dry Nafion membranes observed that spherical ionic clusters are disorderly dispersed on the hydrophobic matrix, and it is difficult to determine what the light and dark regions represent (**Figure** **9a**).^[^ ^153^ ^]^ By RuO_4_ stained treatment, the micro‐phase separation structure was more discernible, with the dark regions representing the ionic clusters and the white regions representing hydrophobic domains (Figure 9b).^[^ ^153^ ^]^ Although heavy‐elements stains (e.g. RuO_4_)^[^ ^153^ ^]^ or ion‐exchange (e.g. Pb^2+^, Cs^+^)^[^ ^154^, ^155^ ^]^ have been successful in enhancing contrast and identifying the ionic clusters and hydrophobic domains in PEMs, the side effects, such as stains induced artifacts, ion‐exchange induced swelling behavior, crystallization behavior of heavy ions during electron‐beam irradiation, will affect the accuracy of phase separation interpretation. Figure 9c showed the SAXS data of bulk Nafion membranes immersed in pure water as well as in aqueous Cu solutions of varying concentrations.^[^ ^156^ ^]^ It can be observed that with the increase of Cu concentration, the ionomer peaks shifted to a higher q region and the ionic domain spacing decreased from 4.6 nm to 3.75 nm, which confirmed that extra ion exchange would affect the inaccuracy of domain size. Regarding this issue, EF‐TEM has been applied to probe the morphology of Nafion membrane based on the subtle difference in the volume plasmon resonance of polymer phases, and the corresponding composite chemical map for ionic clusters (red) and hydrophobic matrix (green) was obtained (Figure 9d,e).^[^ ^156^ ^]^ It is noted that the morphology in the chemical map reflected that observed in the bright‐filed TEM images, and the phase boundaries were more obvious in chemical map images. Further, cryo‐TEM was conducted to study the morphology of hydrated membranes, and more obvious and continuous ionic clusters were observed, which contribute to the high proton conductivity under hydration state (Figure 9f).^[^ ^156^ ^]^ Combining the TEM and cryo‐TEM, the morphology evolution of PEMs from dry to different hydration states can be clearly elucidated, which facilitates the construction of detailed “morphology‐property” relationships.

#### 4.2.2 Scattering Characterization Techniques

**Figure 10:**
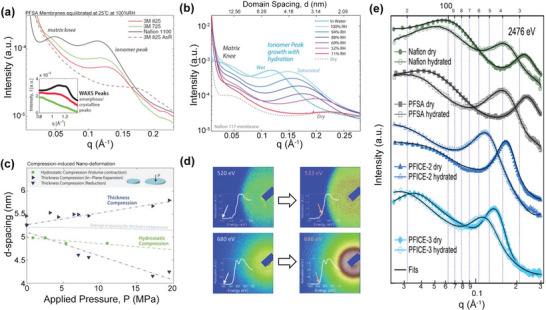
Phase separation structures of PEMs characterized by SAXS and RXS. a) SAXS profiles of three different membranes with the WAXS data shown in the inset,^[^ ^157^ ^]^ b) SAXS profiles of Nafion 117 membrane under different RHs. Reproduced with permission.^[^ ^144^ ^]^ Copyright 2012, American Chemical Society. c) The d‐spacing evolution as a function of pressure for thickness and hydrostatic compression cases. Reproduced with permission.^[^ ^160^ ^]^ Copyright 2012, Elsevier. d) RSoXS images and NEXFS spectra (inset) conducted at different X‐ray energies. Reproduced with permission.^[^ ^147^ ^]^ Copyright 2020, Taylor & Francis. e) Transmission TReXS profiles at 2476 eV of dry and hydrated ionomer membrane. Reproduced with permission.^[^ ^148^ ^]^ Copyright 2019, American Chemical Society.

Except for the direct imaging techniques (mostly TEM), the scattering techniques, including small‐angle X‐ray scattering (SAXS), resonance soft X‐ray scattering (RSoXS), and tender X‐ray scattering (TReXS), have been successful in characterizing phase separation structures of PEMs.^[^ ^146^, ^147^, ^148^, ^157^, ^158^ ^]^ **Figure** **10**a showed the SAXS profiles of three bulk membranes, and two scattering signals can be observed.^[^ ^157^ ^]^ The peaks at *q* = 0.15–0.1 Å^−1^ and *q* = 0.07–0.02 Å^−1^ could be attributed to the ionic clusters and semicrystalline hydrophobic matrix, respectively. The ionomer peak evolution with the PFSA chemistry indicated that increasing the side chain length and decreasing EW could enlarge the ionic cluster size. While for the characteristic peak of matrix knee, it was inapparent in 3M 825 membrane and even disappeared in 3M 725 membrane, which was attributed to the weak crystallization of PFSA backbones (Inset of Figure 10a). Except for the materials, the morphology evolution with humidity is of great significance and has been extensively studied by SAXS.^[^ ^144^, ^159^ ^]^ Figure 10b showed the SAXS profiles of Nafion 117 membrane exposed to different RHs.^[^ ^144^ ^]^ With the increase of RH, the shifted ionomer peak to lower q values, the increased peak intensity, and the decreased full width at half maximum (FWHM) indicated the larger ionic clusters and enhanced short‐range order structure, which could be used to explain the observed hydration‐driven structure‐property relationships. During the operation of PEMFCs, the PEMs often undergo mechanical stretching, including humidity‐induced swelling and external stress‐induced deformation. Thus, the influence of mechanical stretching on morphology and its correlation with PEMs properties are necessary to be studied. Kusoglu et al^[^ ^160^ ^]^ have conducted detailed experiments to study the effect of humidity and external stress on morphology deformation, with the results shown in Figure 10c. It is interesting to note that the *d*‐spacing values of ionic clusters were similar in all directions and increased with increasing humidity, confirming the nano‐structural isotropy of the phase‐separated morphology under different humidities. However, when the external stress was applied in the thickness direction, the anisotropic structures were observed, with the *d*‐spacing of ionic clusters in in‐plane increasing and *d*‐spacing in thickness reducing. Although success has been achieved in morphology characterization by SAXS, the contrast limitation and size probing scale make incomplete structural information difficult in interpreting data.

Resonance soft X‐ray scattering (RSoXS) and tender X‐ray scattering (TReXS) are energy‐tunable and chemically sensitive, providing new opportunities for detailed morphology characterization of PEMs. Oxygen (O) and fluorine (F) are the characteristic elements of PFSA‐based membranes. The RSoXS images and transmission NEXAFS profiles (inset) in Figure 10d indicated that the scattering intensity was amplified when the incident energy was changed from pre‐edge to adsorption edge for both O and F K‐edge, significantly highlighting the semicrystalline domains in the Nafion membranes.^[^ ^147^ ^]^ Further, by conducting the TReXS experiments at the sulfur (S) K‐edge (2476 eV), both the ionomer peak and matrix knee were more obvious, which were easy to be analyzed (Figure 10e).^[^ ^148^ ^]^

### 4.3 Morphology Evolution Process

**Figure 11:**
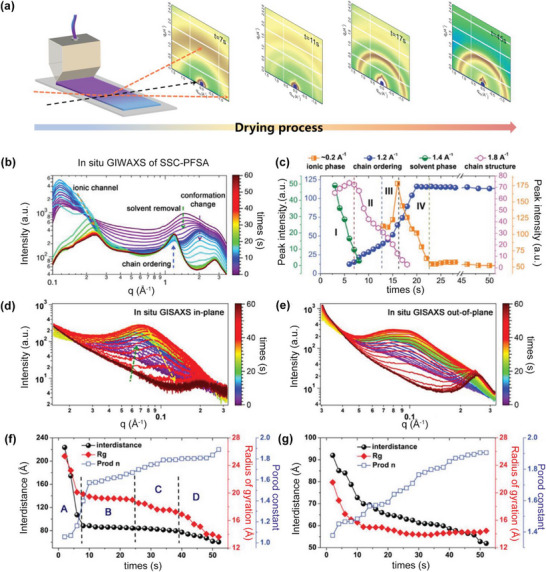
Morphology evolution process characterized by in situ scattering techniques. a) Schematic of set up for in situ characterization. b) In situ GIWAXS profiles of SSC‐PFSA. c) Intensity evolution of characteristic peaks during the drying process. In situ GISAXS profiles in d) in‐plane (IP) and e) out‐of‐plane (OOP) directions. Guinier‐Porod fitting results of GISAXS profiles in f) IP and g) OOP directions. a–g) Reproduced with permission.^[^ ^12^ ^]^ Copyright 2023, American Association for the Advancement of Science.

The unique phase separation structure of PEMs and its strong correlation with properties persuade researchers to investigate the morphology evolution of membranes upon solution casting.^[^ ^12^, ^161^ ^]^ **Figure** **11**a showed the setup for in situ monitoring morphology, in which a slot die printer was used to cast ionomer solution onto a silicon wafer, and then the scattering signals at different times via GIWAXS and GISAXS were collected, respectively. Based on similar equipment, our group conducted the in situ GIWAXS and GISAXS experiments to study the self‐assembly process of SSC‐PFSAs and then extracted a clear picture of the SSC‐PFSA membrane morphology formation.^[^ ^12^ ^]^ Figure 11b showed the in situ GIWAXS profiles, where a few key peaks can be seen and their evolutions with time indicated a distinct structure transformation. The peak at ≈ 1.4 Å^−1^ represented the solvent content. The peak at 1.8–2.0 Å^−1^ showed the change and reorganization of PFSA chain conformation. The peak at 1.2 Å^−1^ was associated with chain order, indicating the crystallization of backbones. The peak at ≈0.2 Å^−1^ represented the formation of ionic clusters. By fitting each characteristic peak, the evolution of peak intensity with time can be extracted (Figure 11c), and the whole process can be divided into four stages. In stage I, the quick evaporation of solvent led to the reorganization of polymer chain into helical perfluorochain conformation. In stage II, the peak intensity of ≈1.2 Å^−1^ increased, and was simultaneously accompanied by a decrease of ≈1.8 Å^−1^, indicating that the ordering of backbones disturbed the specific F‐F in‐chain correlation. In stage III, the further improvement of backbone ordering induced the formation of ionic channels (*q* = 0.2 Å^−1^). In the final stage, a stabilized membrane with fixed ordering (1.2 Å^−1^) and ionic channel packing (0.2 Å^−1^) were achieved. Further, the in situ GISAXS was conducted to probe phase separation of SSC‐PFSA during membrane formation (Figure 11d,e), and the Guinier‐Porod fitting with interference model was applied to analyze the GISAXS results (Figure 11f,g). It can be observed that the aggregates of PFSA nanorods led to the formation of small‐sized ionic clusters, and the further densification of aggregates resulted in the formation of large‐sized ionic channels. The multiscale phase separation structure ensured the continuity of proton channels.

**Figure 12:**
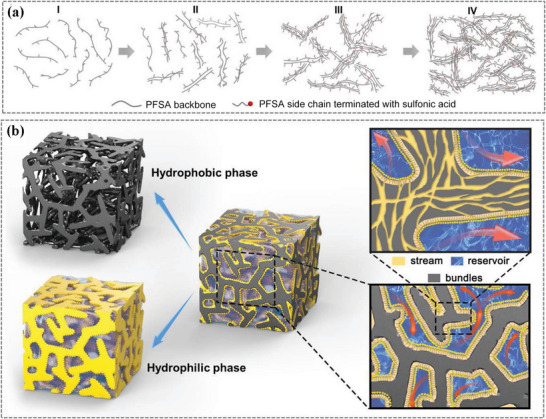
a) Schematic illustration of SSC‐PFSA self‐assembly during the solvent drying process, b) “Steam‐Reservoir” model for PEMs: the grey area represents hydrophobic phase, and the yellow area represents hydrophilic phase. a,b) Reproduced with permission.^[^ ^12^ ^]^ Copyright 2023, American Association for the Advancement of Science.

Combining the in situ GIWAXS and GISAXS results, a picture of morphology evolution process emerged (**Figure** **12**a) and a “stream‐reservoir” model was proposed to describe the membrane morphology (Figure 12b).^[^ ^12^ ^]^ In initial solution state (stage I), the PFSAs are distributed randomly outwards to provide solubility. Then, quick solvent evaporation led to electrostatic‐induced weak aggregates (stage II). Further solvent removal led to a stabilized phase separation and ordered PFSA chains (stages III and IV). The final phase separation morphology can be described by the “stream‐reservoir” model, with “stream” representing the small‐sized ionic clusters (≈2–3 nm) and “reservoir” was the large‐sized void induced by phase separation (≈10 nm) when water swelled. The interconnected steams and reservoirs can trap water quickly under humidity and enable efficient proton transport.

## 5 Summary and Perspective

This review has summarized the crucial advances of HT‐PEMs in fuel cells, with a discussion on the key materials for HT‐PEMs, the degradation mechanisms, the various strategies to improve the performance and durability of HT‐PEMs, and the morphology characterization techniques. The current materials for HT‐PEMs are mainly SA‐containing and PA‐containing materials, where the proton conduction mechanisms and physical‐chemical properties limit the flexibility of operation conditions. For example, the highly humidity‐dependent proton conductivity and the relatively low *T*_g_ determine the modified PFSA membranes and SHPs membranes to operate under <120 °C and humidified conditions, which is not high enough to satisfy the requirements of HT‐PEMFCs. In comparison, the PA‐PBI membranes can work smoothly at 140–200 °C and anhydrous conditions. Despite the success of manipulating the chemical structures or introducing different additives (e.g. inorganic–organic oxides, protic carries, and cross‐linkers) in improving the proton conductivity and fuel cell performance, the performance and durability are still far from the commercial PEMFCs. Moreover, the serious PA leaching when exposed to humidity conditions is another challenge for PA‐PBI membrane to realize its practical applications. The development of novel polymer materials containing strong basic groups, such as QAPOH, PPO, and TB‐based polymers, has led to some breakthroughs in PA‐containing membranes, which not only broaden the operating temperature to 80–200 °C, but also improve the PA tolerant to water.

Despite great advances that have been achieved, there are some challenges to further improving the performance and durability of HT‐PEMs and realizing their practical applications. Future work should focus on the following aspects: 1) Developing new membrane materials to meet the needs of practical applications. In practical applications, the operation of HT‐PEMFCs involves cold start and start‐up/shut‐stop processes, thus determining that the HT‐PEMs should work efficiently and stably under a wide temperature and humidity window. From our perspective, new polymers with both PA and SA groups on the same polymer chains are promising next‐generation materials. The SA‐PA composite proton channels are expected to enable the HT‐PEMFCs to operate under a wide range of temperatures (−30–200 °C) and humidity (0–100% RH). 2) Comprehensive study on the durability of HT‐PEMs and revealing the degradation mechanism. Durability is another important indicator of HT‐PEMFCs. Currently, the durability and degradation mechanism of HT‐PEMs under long‐term high‐temperature conditions are unclear. Thus, more efforts should be devoted to studying the durability of HT‐PEMs and developing efficient strategies to improve their durability.

## Conflict of Interest

The authors declare no conflict of interest.

## References

[1] S. Subianto , M. Pica , M. Casciola , P. Cojocaru , L. Merlo , G. Hards , D. J. Jones , J. Power Sources 2013, 233, 216.

[2] H. Tang , X. Wang , M. Pan , F. Wang , J. Membr. Sci. 2007, 306, 298.

[3] A. Kusoglu , Y. Tang , M. H. Santare , A. M. Karlsson , S. Cleghorn , W. B. Johnson , J. Fuel Cell Sci. Technol. 2008, 6, 011012.

[4] W. Nimir , A. Al‐Othman , M. Tawalbeh , A. Al Makky , A. Ali , H. Karimi‐Maleh , F. Karimi , C. Karaman , Int. J. Hydrogen Energy 2023, 48, 6638.

[5] A. Chandan , M. Hattenberger , A. El‐kharouf , S. Du , A. Dhir , V. Self , B. G. Pollet , A. Ingram , W. Bujalski , J. Power Sources 2013, 231, 264.

[6] Y. Shao , G. Yin , Z. Wang , Y. Gao , J. Power Sources 2007, 167, 235.

[7] J. Zhang , Z. Xie , J. Zhang , Y. Tang , C. Song , T. Navessin , Z. Shi , D. Song , H. Wang , D. P. Wilkinson , Z.‐S. Liu , S. Holdcroft , J. Power Sources 2006, 160, 872.

[8] S. K. Das , A. Reis , K. J. Berry , J. Power Sources 2009, 193, 691.

[9] E. Qu , X. Hao , M. Xiao , D. Han , S. Huang , Z. Huang , S. Wang , Y. Meng , J. Power Sources 2022, 533, 231386.

[10] M. Vinothkannan , A. R. Kim , S. Ramakrishnan , Y.‐T. Yu , D. J. Yoo , Composites Part B 2021, 215, 108828.

[11] C. Yin , J. Li , Y. Zhou , H. Zhang , P. Fang , C. He , ACS Appl. Mater. Interfaces 2018, 10, 14026. 29620850 10.1021/acsami.8b01513

[12] P. Guan , Y. Zou , M. Zhang , W. Zhong , J. Xu , J. Lei , H. Ding , W. Feng , F. Liu , Y. Zhang , Sci. Adv. 2023, 9, eadh1386. 37126562 10.1126/sciadv.adh1386 PMC10132749

[13] P. Xiao , J. Li , R. Chen , R. Wang , M. Pan , H. Tang , Int. J. Hydrogen Energy 2014, 39, 15948.

[14] S. Zhai , Z. Lu , Y. Ai , X. Jia , Y. Yang , X. Liu , M. Tian , X. Bian , J. Lin , S. He , J. Power Sources 2023, 554, 232332.

[15] F. Zhang , Z. Tu , J. Yu , H. Li , C. Huang , H. Zhang , RSC Adv. 2013, 3, 5438.

[16] F. Lu , X. Gao , S. Xie , N. Sun , L. Zheng , Soft Matter 2014, 10, 7819. 25148206 10.1039/c4sm01473a

[17] M. Lei , Y. G. Wang , F. F. Zhang , C. Huang , X. Xu , R. Zhang , D. Y. Fan , Electrochim. Acta 2014, 149, 206.

[18] R. Sood , C. Iojoiu , E. Espuche , F. Gouanvé , G. Gebel , H. Mendil‐Jakani , S. Lyonnard , J. Jestin , J. Phys. Chem. C 2012, 116, 24413.

[19] T.‐H. Kim , T.‐W. Lim , J.‐C. Lee , J. Power Sources 2007, 172, 172.

[20] J. Li , X. Li , Y. Zhao , W. Lu , Z. Shao , B. Yi , ChemSusChem 2012, 5, 896. 22529063 10.1002/cssc.201100725

[21] X. Li , H. Ma , Y. Shen , W. Hu , Z. Jiang , B. Liu , M. D. Guiver , J. Power Sources 2016, 336, 391.

[22] S.‐W. Chuang , S. L.‐C. Hsu , J. Polym. Sci., Part A: Polym. Chem. 2006, 44, 4508.

[23] J. Yang , Y. Xu , L. Zhou , Q. Che , R. He , Q. Li , J. Membr. Sci. 2013, 446, 318.

[24] H. Chen , S. Wang , F. Liu , D. Wang , J. Li , T. Mao , G. Liu , X. Wang , J. Xu , Z. Wang , J. Membr. Sci. 2020, 596, 117722.

[25] D. He , G. Liu , A. Wang , W. Ji , J. Wu , H. Tang , W. Lin , T. Zhang , H. Zhang , J. Membr. Sci. 2022, 650, 120442.

[26] Y. Özdemir , N. Özkan , Y. Devrim , Electrochim. Acta 2017, 245, 1.

[27] X. Li , H. Ma , P. Wang , Z. Liu , J. Peng , W. Hu , Z. Jiang , B. Liu , M. D. Guiver , Chem. Mater. 2020, 32, 1182.

[28] S. Wang , C. Zhao , W. Ma , N. Zhang , Y. Zhang , G. Zhang , Z. Liu , H. Na , J. Mater. Chem. A 2013, 1, 621.

[29] Y. Xiao , Q. Ma , X. Shen , S. Wang , J. Xiang , L. Zhang , P. Cheng , X. Du , Z. Yin , N. Tang , J. Power Sources 2022, 528, 231218.

[30] Y. Xiao , X. Shen , R. Sun , S. Wang , J. Xiang , L. Zhang , P. Cheng , X. Du , Z. Yin , N. Tang , J. Membr. Sci. 2022, 660, 120795.

[31] J. Peng , X. Fu , J. Luo , Y. Liu , L. Wang , X. Peng , J. Membr. Sci. 2022, 643, 120037.

[32] X. Zhang , Q. Liu , L. Xia , D. Huang , X. Fu , R. Zhang , S. Hu , F. Zhao , X. Li , X. Bao , J. Membr. Sci. 2019, 574, 282.

[33] J. Peng , X. Fu , D. Liu , J. Luo , L. Wang , X. Peng , J. Membr. Sci. 2022, 655, 120603.

[34] J. Chen , L. Wang , L. Wang , ACS Appl. Mater. Interfaces 2020, 12, 41350. 32804468 10.1021/acsami.0c10527

[35] Y. Xiao , X. Shen , R. Sun , S. Wang , J. Xiang , L. Zhang , P. Cheng , X. Du , Z. Yin , N. Tang , J. Power Sources 2022, 543, 231802.

[36] P. Wang , J. Lin , Y. Wu , L. Wang , J. Power Sources 2023, 560, 232665.

[37] K.‐S. Lee , J. S. Spendelow , Y.‐K. Choe , C. Fujimoto , Y. S. Kim , Nat. Energy 2016, 1, 16120.

[38] H. Tang , K. Geng , L. Wu , J. Liu , Z. Chen , W. You , F. Yan , M. D. Guiver , N. Li , Nat. Energy 2022, 7, 153.

[39] J. Zhang , S. Chen , H. Wei , J. Zhang , H. Wang , S. Lu , Y. Xiang , Adv. Funct. Mater. 2023, 33, 2214097.

[40] Y. Prykhodko , K. Fatyeyeva , L. Hespel , S. Marais , Chem. Eng. J. 2021, 409, 127329.

[41] O. Danyliv , A. Martinelli , J. Phys. Chem. C 2019, 123, 14813. 10.1021/acs.jpcb.9b01274 30995045

[42] J. Maiti , N. Kakati , S. P. Woo , Y. S. Yoon , Compos. Sci. Technol. 2018, 155, 189.

[43] R. Moore , C. R. Martin , Macromolecules 1989, 22, 3594.

[44] K. D. Kreuer , M. Schuster , B. Obliers , O. Diat , U. Traub , A. Fuchs , U. Klock , S. J. Paddison , J. Maier , J. Power Sources 2008, 178, 499.

[45] N. J. Economou , J. R. O'Dea , T. B. McConnaughy , S. K. Buratto , RSC Adv. 2013, 3, 19525.

[46] P. Guan , J. Lei , X. Liu , K. Xu , S. Pei , H. Ding , Y. Zou , W. Feng , F. Liu , Y. Zhang , Chem. Mater. 2022, 34, 7845.

[47] J. Liu , N. S. Suraweera , D. Keffer , S. Cui , S. Paddison , J. Phys. Chem. C 2010, 114, 11279.

[48] Z. Tu , H. Zhang , Z. Luo , J. Liu , Z. Wan , M. Pan , J. Power Sources 2013, 222, 277.

[49] R. Ding , S. Zhang , Y. Chen , Z. Rui , K. Hua , Y. Wu , X. Li , X. Duan , X. Wang , J. Li , J. Liu , Energy AI 2022, 9, 100170.

[50] A.‐T. Kuo , K. Takeuchi , A. Tanaka , S. Urata , S. Okazaki , W. Shinoda , Polymer 2018, 146, 53.

[51] A. Legala , J. Zhao , X. Li , Energy AI 2022, 13, 100183.

[52] M. E. Günay , N. A. Tapan , Energy AI 2023, 13, 100254.

[53] P. Xiao , J. Li , H. Tang , Z. Wang , M. Pan , J. Membr. Sci. 2013, 442, 65.

[54] P. Guan , J. Lei , Y. Zou , Y. Zhang , Materials 2021, 14, 7875. 34947468 10.3390/ma14247875 PMC8703456

[55] G. Xu , Z. Wu , Z. Wei , W. Zhang , J. Wu , Y. Li , J. Li , K. Qu , W. Cai , Renewable Energy 2020, 153, 935.

[56] G. Xu , Z. Wei , S. Li , J. Li , Z. Yang , S. A. Grigoriev , Int. J. Hydrogen Energy 2019, 44, 29711.

[57] M. Amjadi , S. Rowshanzamir , S. J. Peighambardoust , M. G. Hosseini , M. H. Eikani , Int. J. Hydrogen Energy 2010, 35, 9252.

[58] A. K. Sahu , K. Ketpang , S. Shanmugam , O. Kwon , S. Lee , H. Kim , J. Phys. Chem. C 2016, 120, 15855.

[59] N. J. Steffy , V. Parthiban , A. K. Sahu , J. Membr. Sci. 2018, 563, 65.

[60] G. Rambabu , N. Nagaraju , S. D. Bhat , Chem. Eng. J. 2016, 306, 43.

[61] P. Velayutham , A. K. Sahu , J. Phys. Chem. C 2018, 122, 21735.

[62] V. Ramani , H. R. Kunz , J. M. Fenton , Electrochim. Acta 2005, 50, 1181.

[63] V. Ramani , H. R. Kunz , J. M. Fenton , J. Membr. Sci. 2004, 232, 31.

[64] C. del Río , E. Morales , P. G. Escribano , Int. J. Hydrogen Energy 2014, 39, 5326.

[65] C. Schmidt , T. Glück , G. Schmidt‐Naake , Chem. Eng. Technol. 2008, 31, 13.

[66] F. Lu , X. Gao , X. Yan , H. Gao , L. Shi , H. Jia , L. Zheng , ACS Appl. Mater. Interfaces 2013, 5, 7626. 23855417 10.1021/am401940y

[67] Y. Li , Y. Shi , N. Mehio , M. Tan , Z. Wang , X. Hu , G. Z. Chen , S. Dai , X. Jin , Appl. Energy 2016, 175, 451.

[68] S. Akbari , M. T. Hamed Mosavian , F. Moosavi , A. Ahmadpour , Composites, Part B 2019, 161, 402.

[69] Y. Liu , S. V. Sambasivarao , J. L. Horan , Y. Yang , C. M. Maupin , A. M. Herring , J. Phys. Chem. C 2013, 118, 854.

[70] S. V. Sambasivarao , Y. Liu , J. L. Horan , S. Seifert , A. M. Herring , C. M. Maupin , J. Phys. Chem. C 2014, 118, 20193.

[71] V. M. Ortiz‐Martínez , A. Ortiz , V. Fernández‐Stefanuto , E. Tojo , M. Colpaert , B. Améduri , I. Ortiz , Polymer 2019, 179, 121583.

[72] H. Q. Li , X. J. Liu , J. Xu , D. Xu , H. Ni , S. Wang , Z. Wang , J. Membr. Sci. 2016, 509, 173.

[73] R. Rath , P. Kumar , L. Unnikrishnan , S. Mohanty , S. K. Nayak , Polym. Rev. 2019, 60, 267.

[74] K. Jiao , J. Xuan , Q. Du , Z. Bao , B. Xie , B. Wang , Y. Zhao , L. Fan , H. Wang , Z. Hou , S. Huo , N. P. Brandon , Y. Yin , M. D. Guiver , Nat. 2021, 595, 361. 10.1038/s41586-021-03482-7 34262215

[75] L. Zhao , Y. Li , H. Zhang , W. Wu , J. Liu , J. Wang , J. Power Sources 2015, 286, 445.

[76] S. Qu , C. Zhang , M. Li , Y. Zhang , L. Chen , Y. Yang , B. Kang , Y. Wang , J. Duan , W. Wang , Korean J. Chem. Eng. 2019, 36, 2125.

[77] K. T. Park , S. G. Kim , J. H. Chun , D. H. Jo , B.‐H. Chun , W. I. Jang , G. B. Kang , S. H. Kim , K. B. Lee , Int. J. Hydrogen Energy 2011, 36, 10891.

[78] S. M. J. Zaidi , S. D. Mikhailenko , G. P. Robertson , M. D. Guiver , S. Kaliaguine , J. Membr. Sci. 2000, 173, 17.

[79] H. Zhang , W. Wu , J. Wang , T. Zhang , B. Shi , J. Liu , S. Cao , J. Membr. Sci. 2015, 476, 136.

[80] Q. Che , R. He , J. Yang , L. Feng , R. F. Savinell , Electrochem. Commun. 2010, 12, 647.

[81] P. R. Jothi , S. Dharmalingam , J. Membr. Sci. 2014, 450, 389.

[82] V. Elumalai , T. Ganesh , C. Selvakumar , D. Sangeetha , Mater. Sci. Energy Technol. 2018, 1, 196.

[83] N. R. Kang , T. H. Pham , P. Jannasch , ACS Macro Lett. 2019, 8, 1247. 35651144 10.1021/acsmacrolett.9b00615

[84] K. Miyatake , H. Zhou , H. Uchida , M. Watanabe , Chem. Commun. (Camb) 2003, 368. 12613616 10.1039/b210296j

[85] Y. Chikashige , Y. Chikyu , K. Miyatake , M. Watanabe , Macromolecules 2005, 38, 7121.

[86] Y. Kozawa , S. Suzuki , M. Miyayama , T. Okumiya , E. Traversa , Solid State Ionics 2010, 181, 348.

[87] I. Colicchio , F. Wen , H. Keul , U. Simon , M. Moeller , J. Membr. Sci. 2009, 326, 45.

[88] X. Wang , M. Jin , Y. Li , L. Zhao , Electrochim. Acta 2017, 257, 290.

[89] B. Zhang , Y. Cao , Z. Li , H. Wu , Y. Yin , L. Cao , X. He , Z. Jiang , Electrochim. Acta 2017, 240, 186.

[90] R. Haider , Y. Wen , Z. F. Ma , D. P. Wilkinson , L. Zhang , X. Yuan , S. Song , J. Zhang , Chem. Soc. Rev. 2021, 50, 1138. 33245736 10.1039/d0cs00296h

[91] K. Hooshyari , M. Javanbakht , M. Adibi , Int. J. Hydrogen Energy 2016, 41, 10870.

[92] Y. L. Ma , J. S. Wainright , M. H. Litt , R. F. Savinell , J. Electrochem. Soc. 2004, 151, A8.

[93] Z. Guo , M. Perez‐Page , J. Chen , Z. Ji , S. M. Holmes , J. Energy Chem. 2021, 63, 393.

[94] C. Xu , X. Liu , J. Cheng , K. Scott , J. Power Sources 2015, 274, 922.

[95] J. Yang , C. Liu , L. Gao , J. Wang , Y. Xu , R. He , RSC Adv. 2015, 5, 101049.

[96] C. Xu , Y. Cao , R. Kumar , X. Wu , X. Wang , K. Scott , J. Mater. Chem. 2011, 21, 11359.

[97] S. Singha , T. Jana , ACS Appl. Mater. Interfaces 2014, 6, 21286. 25365766 10.1021/am506260j

[98] S. Maity , S. Singha , T. Jana , Polymer 2015, 66, 76.

[99] Q. Liu , X. Wang , X. Zhang , Z. Ling , W. Wu , X. Fu , R. Zhang , S. Hu , X. Li , F. Zhao , X. Bao , J. Cleaner Prod. 2022, 359, 131977.

[100] J. Peng , P. Wang , B. Yin , X. Fu , L. Wang , J. Luo , X. Peng , J. Membr. Sci. 2021, 640, 119775.

[101] C. Lee , H. Na , Y. Jeon , H. Jung Hwang , H.‐J. Kim , I. Mochida , S.‐H. Yoon , J.‐I. Park , Y.‐G. Shul , J. Ind. Eng. Chem. 2019, 74, 7.

[102] Y. Chen , F. Xu , J. Li , Y. Han , J. Qiao , J. Liu , Y. Xu , B. Lin , ACS Appl. Energy Mater. 2023, 6, 2594.

[103] H. Bai , H. Peng , Y. Xiang , J. Zhang , H. Wang , S. Lu , L. Zhuang , J. Power Sources 2019, 443, 227219.

[104] Y. Bai , D. Han , M. Xiao , Z. Huang , C. Wang , S. Wang , Y. Meng , J. Power Sources 2023, 563, 232823.

[105] Y. Jin , T. Wang , X. Che , J. Dong , R. Liu , J. Yang , J. Membr. Sci. 2022, 641, 119884.

[106] H. Bai , H. Wang , J. Zhang , J. Zhang , S. Lu , Y. Xiang , J. Membr. Sci. 2019, 592, 117395.

[107] J. Zhang , J. Zhang , H. Bai , Q. Tan , H. Wang , B. He , Y. Xiang , S. Lu , J. Membr. Sci. 2019, 572, 496.

[108] J. Wu , X. Z. Yuan , J. J. Martin , H. Wang , J. Zhang , J. Shen , S. Wu , W. Merida , J. Power Sources 2008, 184, 104.

[109] R. M. H. Khorasany , A. Sadeghi Alavijeh , E. Kjeang , G. G. Wang , R. K. N. D. Rajapakse , J. Power Sources 2015, 274, 1208.

[110] C. A. Wilkie , J. R. Thomsen , M. L. Mittleman , J. Appl. Polym. Sci. 1991, 42, 901.

[111] L. Ghassemzadeh , K. D. Kreuer , J. Maier , K. Müller , J. Power Sources 2011, 196, 2490.

[112] Z. Rui , J. Liu , Prog. Nat. Sci.: Mater. Int. 2020, 30, 732.

[113] E. Endoh , S. Terazono , H. Widjaja , Y. Takimoto , Electrochem. Solid‐State Lett. 2004, 7, A209.

[114] M. Robert , A. El Kaddouri , J.‐C. Perrin , J. Raya , O. Lottin , J. Membr. Sci. 2021, 621, 118977.

[115] M Wakizoe , H Murata , H Takei , Proc. Fuel Cell Sem., Portland, USA 1998, 487.

[116] H. Xu , M. Wu , Y. Liu , V. Mittal , F. Kassim , B. Vieth , L. Bonville , H. R. Kunz , J. M. Fenton , ECS Trans. 2006, 3, 561.

[117] B. Lv , K. Geng , H. Yin , C. Yang , J. Hao , Z. Luan , Z. Huang , X. Qin , W. Song , N. Li , Z. Shao , J. Membr. Sci. 2021, 639, 119760.

[118] J. Hao , Y. Jiang , X. Gao , F. Xie , Z. Shao , B. Yi , J. Membr. Sci. 2017, 522, 23.

[119] Z. Wang , H. Tang , H. Zhang , M. Lei , R. Chen , P. Xiao , M. Pan , J. Membr. Sci. 2012, 421, 201.

[120] S. Park , H. Lee , S. H. Shin , N. Kim , D. Shin , B. Bae , ACS Omega 2018, 3, 11262. 31459234 10.1021/acsomega.8b01063 PMC6644771

[121] Y. Park , D. Kim , J. Membr. Sci. 2018, 566, 1.

[122] V. Prabhakaran , C. G. Arges , V. Ramani , Proc. Natl. Acad. Sci. USA 2012, 109, 1029. 22219367 10.1073/pnas.1114672109 PMC3268320

[123] Q. Li , C. Pan , J. O. Jensen , P. Noyé , N. J. Bjerrum , Chem. Mater. 2007, 19, 350.

[124] Q. Li , Solid State Ionics 2004, 168, 177.

[125] S. Yu , L. Xiao , B. C. Benicewicz , Fuel Cells 2008, 8, 165.

[126] A. S. Lee , Y.‐K. Choe , I. Matanovic , Y. S. Kim , J. Mater. Chem. A 2019, 7, 9867.

[127] S. S. Araya , F. Zhou , V. Liso , S. L. Sahlin , J. R. Vang , S. Thomas , X. Gao , C. Jeppesen , S. K. Kær , Int. J. Hydrogen Energy 2016, 41, 21310.

[128] P. Sun , Z. Li , S. Wang , X. Yin , J. Membr. Sci. 2018, 549, 660.

[129] M. Hu , T. Li , S. Neelakandan , L. Wang , Y. Chen , J. Membr. Sci. 2020, 593, 117435.

[130] Q. F. Li , H. C. Rudbeck , A. Chromik , J. O. Jensen , C. Pan , T. Steenberg , M. Calverley , N. J. Bjerrum , J. Kerres , J. Membr. Sci. 2010, 347, 260.

[131] Y. Zhai , H. Zhang , Y. Zhang , D. Xing , J. Power Sources 2007, 169, 259.

[132] Y.‐C. Cao , C. Xu , L. Zou , K. Scott , J. Liu , J. Power Sources 2015, 294, 691.

[133] A. Kusoglu , A. Z. Weber , Chem. Rev. 2017, 117, 987. 28112903 10.1021/acs.chemrev.6b00159

[134] S. Y. Choi , M. M. Ikhsan , K. S. Jin , D. Henkensmeier , Int. J. Energy Res. 2022, 46, 11265.

[135] J. Song , M. Zhang , M. Yuan , Y. Qian , Y. Sun , F. Liu , Small Methods 2018, 2, 1700229.

[136] J. Wang , M. Yang , P. Dou , X. Wang , H. Zhang , Ind. Eng. Chem. Res. 2014, 53, 14175.

[137] K. Feng , L. Hou , B. Tang , P. Wu , Phys. Chem. Chem. Phys. 2015, 17, 9106. 25759084 10.1039/c5cp00203f

[138] K. Lee , A. Ishihara , S. Mitsushima , N. Kamiya , K. Ota , J. Electrochem. Soc. 2004, 151, A639.

[139] G. M. Divoux , K. A. Finlay , J. K. Park , J.‐M. Song , B. Yan , M. Zhang , D. A. Dillard , R. B. Moore , ECS Trans. 2011, 41, 87.

[140] T. Takamatsu , A. Eisenberg , J. Appl. Polym. Sci. 1979, 24, 2221.

[141] R. B. Moore , C. R. Martin , Macromolecules 1989, 22, 3594.

[142] P. C. van der Heijden , L. Rubatat , O. Diat , Macromolecules 2004, 37, 5327.

[143] H. Mendil‐Jakani , S. Pouget , G. Gebel , P. N. Pintauro , Polymer 2015, 63, 99.

[144] A. Kusoglu , S. Savagatrup , K. T. Clark , A. Z. Weber , Macromolecules 2012, 45, 7467.

[145] G. Gebel , P. Aldebert , M. Pineri , Macromolecules 1987, 20, 1425.

[146] A. Kusoglu , K. Vezzù , G. A. Hegde , G. Nawn , A. R. Motz , H. N. Sarode , G. M. Haugen , Y. Yang , S. Seifert , M. A. Yandrasits , S. Hamrock , C. M. Maupin , A. Z. Weber , V. Di Noto , A. M. Herring , Chem. Mater. 2019, 32, 38.

[147] G. M. Su , I. A. Cordova , C. Wang , Synchrotron Radiat. News 2020, 33, 17.

[148] G. M. Su , I. A. Cordova , M. A. Yandrasits , M. Lindell , J. Feng , C. Wang , A. Kusoglu , J. Am. Chem. Soc. 2019, 141, 13547. 31430144 10.1021/jacs.9b05322

[149] C. Wang , V. Krishnan , D. Wu , R. Bledsoe , S. J. Paddison , G. Duscher , J. Mater. Chem. A 2013, 1, 938.

[150] S.‐X. Zhao , L.‐J. Zhang , Y.‐X. Wang , J. Power Sources 2013, 233, 309.

[151] S. Ryu , J.‐H. Kim , J.‐Y. Lee , S.‐H. Moon , J. Mater. Chem. A 2018, 6, 20836.

[152] T. Makinouchi , M. Tanaka , H. Kawakami , J. Membr. Sci. 2017, 530, 65.

[153] T. Xue , J. S. Trent , K. Osseo‐Asare , J. Membr. Sci. 1989, 45, 261.

[154] T. D. Gierke , G. E. Munn , F. C. Wilson , J. Polym. Sci., Polym. Phys. Ed. 1981, 19, 1687.

[155] Y.‐M. Kim , K.‐W. Park , J.‐H. Choi , I.‐S. Park , Y.‐E. Sung , Electrochem. Commun. 2003, 5, 571.

[156] F. I. Allen , L. R. Comolli , A. Kusoglu , M. A. Modestino , A. M. Minor , A. Z. Weber , ACS Macro Lett. 2015, 4, 1. 35596390 10.1021/mz500606h

[157] A. Kusoglu , T. J. Dursch , A. Z. Weber , Adv. Funct. Mater. 2016, 26, 4961.

[158] W. Zhong , F. Liu , C. Wang , J. Phys.: Condens. Matter 2021, 33, 313001. 10.1088/1361-648X/ac0194 34140434

[159] G. Gebel , Polymer 2000, 41, 5829.

[160] A. Kusoglu , A. Hexemer , R. Jiang , C. S. Gittleman , A. Z. Weber , J. Membr. Sci. 2012, 421–422, 283.

[161] P. J. Dudenas , A. Kusoglu , Macromolecules 2019, 52, 7779.

